# Peach genetic resources: diversity, population structure and linkage disequilibrium

**DOI:** 10.1186/1471-2156-14-84

**Published:** 2013-09-16

**Authors:** Xiong-wei Li, Xian-qiao Meng, Hui-juan Jia, Ming-liang Yu, Rui-juan Ma, Li-rong Wang, Ke Cao, Zhi-jun Shen, Liang Niu, Jian-bao Tian, Miao-jin Chen, Ming Xie, Pere Arus, Zhong-shan Gao, Maria Jose Aranzana

**Affiliations:** 1 Department of Horticulture, Key Laboratory for Horticultural Plant Growth, Development and Quality Improvement of State Agriculture Ministry, Zhejiang University, Hangzhou 310058, China; 2 Horticultural Institute, Jiangsu Academy of Agricultural Sciences, Zhong-Lin Street 50, Nanjing 210014, China; 3 Zhenzhou Fruit Research Institute, CAAS, Zhengzhou China; 4 Pomology Institute, Shanxi Academy of Agricultural Sciences, Ke Yuan Road 1, Taigu, Shanxi 030815, China; 5 Fenghua Honey Peach Institute, Xikou, Fenghua, Zhejiang Province 315521, China; 6 Institute of Horticulture, Zhejiang Academy of Agricultural Sciences, Hangzhou 310021, China; 7 IRTA. Centre de Recerca en Agrigenòmica CSIC-IRTA-UAB, Campus UAB – Edifici CRAG, Bellaterra - Cerdanyola del Vallès, 08193 Barcelona, Spain

## Abstract

**Background:**

Peach (*Prunus persica* (L.) Batsch) is one of the most important model fruits in the Rosaceae family. Native to the west of China, where peach has been domesticated for more than 4,000 years, its cultivation spread from China to Persia, Mediterranean countries and to America. Chinese peach has had a major impact on international peach breeding programs due to its high genetic diversity. In this research, we used 48 highly polymorphic SSRs, distributed over the peach genome, to investigate the difference in genetic diversity, and linkage disequilibrium (LD) among Chinese cultivars, and North American and European cultivars, and the evolution of current peach cultivars.

**Results:**

In total, 588 alleles were obtained with 48 SSRs on 653 peach accessions, giving an average of 12.25 alleles per locus. In general, the average value of observed heterozygosity (0.47) was lower than the expected heterozygosity (0.60). The separate analysis of groups of accessions according to their origin or reproductive strategies showed greater variability in Oriental cultivars, mainly due to the high level of heterozygosity in Chinese landraces. Genetic distance analysis clustered the cultivars into two main groups: one included four wild related *Prunus*, and the other included most of the Oriental and Occidental landraces and breeding cultivars. STRUCTURE analysis assigned 469 accessions to three subpopulations: Oriental (234), Occidental (174), and Landraces (61). Nested STRUCTURE analysis divided the Oriental subpopulation into two different subpopulations: ‘Yu Lu’ and ‘Hakuho’. The Occidental breeding subpopulation was also subdivided into nectarine and peach subpopulations. Linkage disequilibrium (LD) analysis in each of these subpopulations showed that the percentage of linked (r^2^ > 0.1) intra-chromosome comparisons ranged between 14% and 47%. LD decayed faster in Oriental (1,196 Kbp) than in Occidental (2,687 Kbp) samples. In the ‘Yu Lu’ subpopulation there was considerable LD extension while no variation of LD with physical distance was observed in the landraces. From the first STRUCTURE result, LG1 had the greatest proportion of alleles in LD within all three subpopulations.

**Conclusions:**

Our study demonstrates a high level of genetic diversity and relatively fast decay of LD in the Oriental peach breeding program. Inclusion of Chinese landraces will have a greater effect on increasing genetic diversity in Occidental breeding programs. Fingerprinting with genotype data for all 658 cultivars will be used for accession management in different germplasms. A higher density of markers are needed for association mapping in Oriental germplasm due to the low extension of LD. Population structure and evaluation of LD provides valuable information for GWAS experiment design in peach.

## Background

Peach (*Prunus persica* (L) Batsch) is one of the most predominant commercially grown stone fruits in the Rosaceae family, subfamily Spiroideae because of its broad climate adaptation and high production in cultivation regions [1]. Its short juvenile period (2–3 years) and the ease of obtaining controlled crosses have made peach breeding programs quite successful: around 1,000 new cultivars were released during 1991–2001 [2]. In addition, because of its small genome size and the simple genetic basis of many morphological and economical traits [3], peach is a model fruit crop for traditional genetics and current genomics research, with subsequent applications in breeding and selection.

Being the centre of origin of peach, China has the longest history of peach cultivation (more than 4,000 years), and the richness of genetically diverse germplasm can provide useful genes to breed cultivars with enhanced resistance to pests and diseases, improved fruit size and quality, and a longer postharvest shelf-life. The ancestral form peach used as rootstock in south China still exists. Other wild related species are present in the north-western region of China: ‘*P. mira* Koehne’ , ‘*P. kansuensis.* Rehd’ , ‘*P. davidiana.* Franch’ and ‘*P. potaninii* Batal’. In China, the main peach germplasms are in three national collections, but regional and local collections are also established around the country. The national collections preserve 2,000 accessions from China and foreign countries, with about 600 cultivars of local origin [4]. Based on genetic fingerprint data, Chinese peach cultivars have more genetic diversity than has been reported for other peach germplasm collections [5]. The Chinese peach germplasm has had a great impact on breeding research in other countries. After introducing ‘Shanghai Shui Mi’ as parents in the early 20^th^ century, Japan selected out ‘Hakuto’ [6,7] and the USA released the famous cultivar ‘Elberta’. Both ‘Hakuto’ and ‘Elberta’ were extensively used as parents for further breeding of modern cultivars [8,9]. Over the last few decades, considerable effort has been put into peach breeding in the USA, South Africa, Brazil, Argentina, Australia, China, Spain, Italy, France and Japan [10], producing almost 2,000 new cultivars; half of these have been registered in and come from the USA while only 5% are from China [11,12].

As a self-pollinated species, peach retains a high degree of self-compatibility and homozygosity [13]. During the decade 1991–2001, peach and nectarine cultivars were generated through controlled crosses (43-61%), open pollination (15-21%) and bud mutation (4-5%), and the outcrossing range varied from 15 to 30% [14]. Most local Spanish varieties were self propagated; melting cultivars were usually produced by crossing two individuals and selecting from their progeny, and non-melting peaches were selected from seed-propagated populations [15]. Chinese breeding cultivars were mainly released using ‘Shanghai Shui Mi’(‘Chinese Cling’) and ‘Bai Hua Shui Mi’ as founders. ‘Okubo’ and ‘Hakuto’ from Japan were inter-crossed to produce white and low-acid peaches in Nanjing and Beijing germplasms. ‘NJN76’ , ‘Mayfire’ and ‘Legrant’ were also introduced and inter-crossed to produce nectarines. Chinese landrace reproduction was mainly based on seed propagation [4]. Most of the Japanese peaches were selections or mutations of ‘Hakuto’ and ‘Hakuho’ [6,7]. New genetic backgrounds should be explored and introduced in peach breeding programs to overcome the narrow genetic background resulting from the use of few founders [15-20].

Peach genetics and genomics studies have provided tools for marker-assisted selection (MAS). Microsatellites and simple sequence repeat markers (SSRs) have proved to be a very efficient way to evaluate genetic relationships between individuals, marker-assisted selections and for population genetics studies in *Prunus* species [15-20]. Today, approximately 500 SSRs have been mapped in the reference map (T × E), and more microsatellites are available from the complete peach genome sequence data produced by the International Peach Genome Initiative (IPGI) [21] ( http://www.rosaceae.org/species/prunus_persica/genome_v1.0).

Linkage disequilibrium (LD) mapping (also known as association mapping) is a gene mapping tool which relies on the association between molecular markers and phenotypic traits in populations of unrelated individuals; crosses are not always available or easy to obtain. The extent of linkage disequilibrium around a gene has major implications in association mapping, since it determines the effectiveness of this approach [22]. Low LD implies a high number of markers, whereas very high LD extension means low mapping resolution [23]. A high level of information on LD patterns in the working species, in our case peach, is needed for any further association mapping studies. Whole-genome LD can be confusing if the sample is structured into subpopulations (also known as population stratification), i.e. when two (or a group of) accessions have a higher probability of sharing the same allele due to their origin (geography, breeding program, etc.) [24].

Different factors can increase the level of LD: small population size, inbreeding, genetic isolation between lineages, population subdivision, low recombination rate, population admixture, genetic drift and epistasis. In contrast, outcrossing, high recombination rate, high mutation rate and gene conversion can decrease the LD [22,24]. The amount, extent and distribution of LD have been well described for common human diseases. In plants, LD has also been investigated in maize, barley, ryegrass, wheat, soybean, sugarcane, grapevine and peach, to design association-mapping experiments and infer the evolution of species [25-27].

Up to now, variability and LD analyses have been reported separately in Occidental and Oriental peach collections [15,28]. High levels of LD, extending to 13–15 cM, have been reported in American and European peach accessions, and the development of the whole-genome scanning approach for genetic studies has also been raised [15]. The LD level in Chinese landraces has been reported to span 6.01 cM [28]. So far there has been no report on the comparison of genetic diversity, population structure and LD between Oriental and Occidental accessions using the same markers and analytical methods. Moreover, although several reports from China have dealt with it using a limited number of accessions and SSR markers [18,19], a complete picture of Oriental peach genetic diversity and population structure is lacking.

In this research, we investigated the genetic diversity, population structure and linkage disequilibrium of a large group of heterogeneous samples of Oriental accessions and integrated the data with that obtained in [15] to analyse, jointly, 653 peach accessions. We used this comparison between Oriental and Occidental germplasms to infer how genetic diversity can be increased by combining both sets of collections, and provide guidance for introducing accessions into different germplasms.

## Results

### Genetic diversity of the accessions

The 48 SSRs selected in this research were polymorphic in both Oriental and Occidental samples, amplifying a total of 588 alleles (Table 1), with an average of 12.25 alleles per locus. The frequency of most of the alleles (435, 73.9%) was less than 5%, and the frequency of 114 alleles was less than 1%. Low allele frequencies resulted in a low effective number of alleles (2.93). The observed heterozygosity (Ho) ranged from 0.13 (PMS02) to 0.63 (UDP96-005), with an average of 0.47. These were lower than the expected values (He), which ranged from 0.17 (PMS02) to 0.85 (BPPCT006), an average of 0.60. Consequently, Wright’s fixation indices (F) were positive. As expected, loci were highly informative: the highest power of discrimination (PD) between two random cultivars was observed in BPPCT006 (PD = 0.95), and the lowest in PMS02 (PD = 0.28). The number of genotypes for each locus varied from seven (pchgms1) to 65 (BPPCT006).

**Table 1:** Diversity parameters of the SSRs for the tested peach cultivars

**Marker**	**Sample size**	**Ao**	**Ae**	**Ho**	**He**	**F**	**PD**	**MAF**	**Gn**
**UDP96-018**	653	12	2.33	0.42	0.55	0.25	0.73	0.63	23
**CPPCT027**	653	11	2.32	0.46	0.50	0.08	0.68	0.68	22
**EPPCU1090**	653	7	2.70	0.53	0.59	0.10	0.79	0.59	14
**UDP96-005**	653	15	4.31	0.63	0.73	0.14	0.88	0.41	41
**CPPCT026**	653	21	5.01	0.61	0.78	0.23	0.90	0.37	56
**BPPCT020**	653	10	2.59	0.49	0.65	0.24	0.81	0.43	16
**CPPCT029**	653	13	2.27	0.45	0.61	0.26	0.77	0.50	34
**CPPCT044**	653	13	4.30	0.52	0.76	0.31	0.89	0.41	39
**CPPCT042**	653	15	2.19	0.41	0.64	0.36	0.80	0.50	33
**BPPCT001**	653	14	3.11	0.51	0.79	0.35	0.90	0.36	47
**BPPCT024**	653	10	5.52	0.49	0.75	0.35	0.85	0.42	28
**UDP96-013**	653	14	3.73	0.50	0.76	0.34	0.88	0.31	31
**pceGA34**	653	9	2.05	0.41	0.55	0.26	0.72	0.51	16
**pchgms1**	653	6	1.71	0.23	0.31	0.26	0.46	0.81	7
**UDP98-025**	653	8	2.87	0.47	0.75	0.38	0.88	0.35	21
**BPPCT007**	653	11	4.34	0.63	0.73	0.15	0.88	0.43	33
**BPPCT039**	653	7	1.61	0.41	0.39	−0.05	0.57	0.75	11
**CPPCT002**	653	8	2.37	0.42	0.59	0.28	0.74	0.46	14
**UDP96-008**	653	8	2.82	0.47	0.61	0.23	0.79	0.52	18
**UDP96-003**	653	15	2.99	0.61	0.66	0.08	0.80	0.43	40
**UDP98-024**	653	11	2.56	0.56	0.63	0.12	0.81	0.43	19
**PTS1-SSR**	429	10	3.95	0.61	0.75	0.18	0.89	0.34	21
**CPPCT005**	653	15	2.31	0.48	0.61	0.21	0.79	0.54	26
**BPPCT015**	653	20	2.82	0.55	0.66	0.17	0.81	0.46	43
**CPPCT046**	653	12	2.69	0.44	0.62	0.29	0.78	0.45	22
**CPPCT040**	653	12	2.68	0.58	0.63	0.07	0.76	0.51	25
**EPPCU1775**	429	13	3.68	0.51	0.73	0.30	0.75	0.39	26
**UDP97-401**	653	11	2.25	0.45	0.52	0.14	0.67	0.58	16
**BPPCT017**	653	16	3.95	0.58	0.71	0.18	0.86	0.38	44
**BPPCT037**	653	16	1.92	0.49	0.50	0.02	0.66	0.66	25
**BPPCT038**	653	13	3.28	0.52	0.69	0.24	0.83	0.48	40
**BPPCT014**	653	11	1.55	0.28	0.36	0.21	0.50	0.79	16
**CPPCT013**	653	6	1.64	0.28	0.29	0.05	0.46	0.83	11
**CPSCT006**	653	10	2.05	0.40	0.42	0.05	0.61	0.74	20
**CPPCT015**	653	13	1.46	0.16	0.23	0.34	0.34	0.87	30
**BPPCT025**	653	16	5.54	0.62	0.81	0.24	0.94	0.30	56
**pchcms5**	653	10	2.41	0.46	0.53	0.13	0.72	0.64	20
**UDP96-001**	653	13	4.04	0.51	0.68	0.26	0.84	0.50	30
**CPPCT022**	653	24	3.77	0.59	0.78	0.25	0.92	0.35	58
**CPPCT030**	653	14	2.28	0.56	0.60	0.07	0.79	0.58	34
**CPPCT033**	653	9	1.84	0.24	0.56	0.57	0.68	0.58	19
**PMS02**	653	12	1.23	0.13	0.17	0.23	0.28	0.91	22
**pchgms6**	653	14	3.17	0.49	0.70	0.30	0.86	0.42	35
**BPPCT006**	653	15	6.97	0.63	0.85	0.26	0.95	0.26	65
**UDP98-409**	653	13	2.80	0.49	0.60	0.19	0.77	0.57	24
**CPPCT006**	653	10	2.25	0.49	0.63	0.23	0.79	0.49	15
**PCeGA25**	429	8	1.61	0.36	0.38	0.06	0.54	0.76	13
**Pchgms3**	653	14	2.88	0.48	0.57	0.17	0.74	0.61	29
**Mean**		12.25	2.93	0.47	0.60	0.22	0.75	0.53	28

Note: *Ao* number of observed alleles, *Ae* effective number of alleles, *Ho* observed heterozygosity, *He* expected heterozygosity.

*MAF* frequency of major allele, *F* Wright’s fixation index, *PD* power of discrimination, *Gn* number of genotype at each locus.

**Figure 1:**
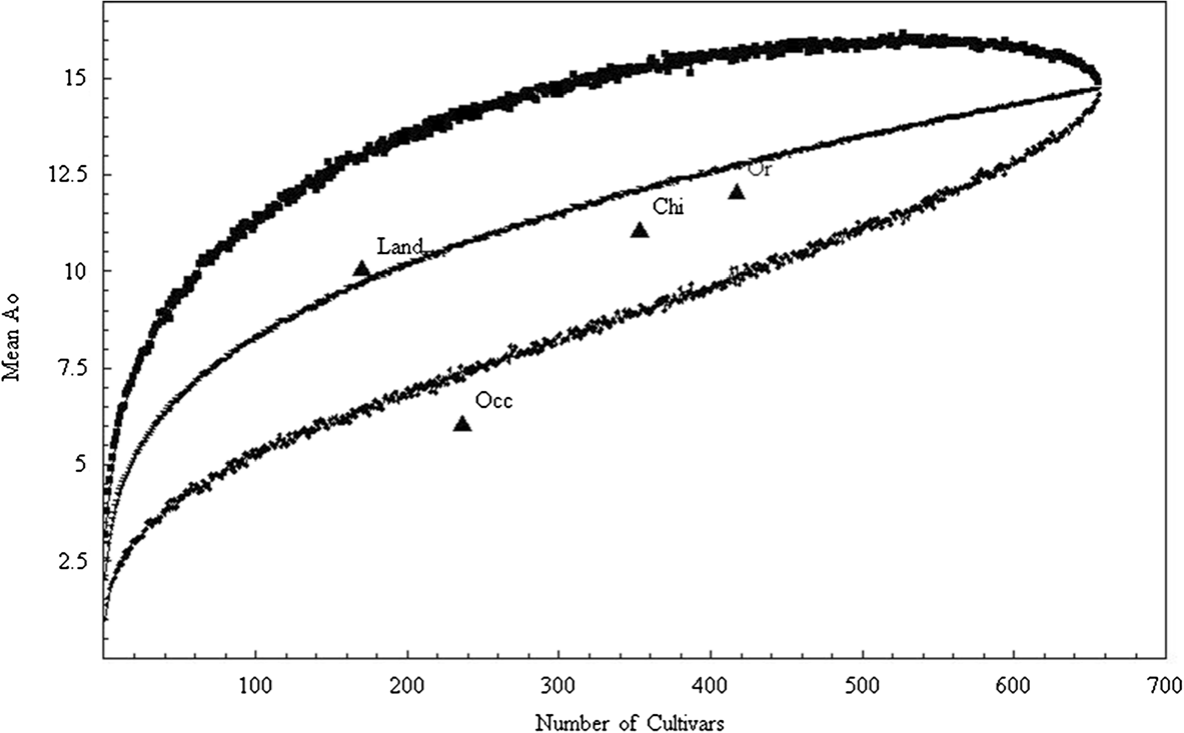
**Rarefaction curve of mean number of observed alleles in sample subsets of different sample size.** The values for the number of alleles observed in Oriental (Or), Occidental (Occ), Chinese (Chi) and landraces (Land).

The sample of accessions studied was highly heterogeneous, covering different geographic regions (roughly split as Oriental and Occidental) as well as different degrees of domestication (wild, landraces and breeding). In order to explore and compare the variability inherent in such heterogeneity, variability parameters were calculated in 12 sample subdivisions (Table 2). Twice the number of alleles (12 versus 6) was amplified in the Oriental group than in the Occidental group. Due to the large differences in sample size, the evaluation of the mean number of observed alleles in 4 groups was plotted, with increasing sample size. Figure 1 demonstrates that the Oriental accessions (with number of observed alleles close to the mean on the standard curve) contributed more than the Occidental accessions (with number of alleles below the 95% CI of the distribution) to the variability of the whole collection. The observed heterozygosity was higher in the Oriental group than that in the Occidental group (0.53 versus 0.35), while the deviation from the expected heterozygosity was lower than in the Occidental samples, within which most samples come from breeding programs and directed crosses, 0.53 vs 0.61 (Ho vs He) in the Oriental group and 0.35 vs 0.47 in the Occidental group.

**Table 2:** Genetic diversity for different peach cultivar subsets based on 48 SSRs

**Subset of accessions**	**Sample Size**	**Ao**	**Ae**	**Ho**	**He**	**F**
All cultivars	653	12.25	2.93	0.47	0.60	0.22
Oriental	417	12	2.93	0.53	0.61	0.13
Japanese + Korea	64	5	2.56	0.56	0.53	−0.03
Chinese	353	11	2.99	0.53	0.61	0.14
Chinese cultivars	207	8	2.75	0.53	0.58	0.10
Chinese landraces	146	10	3.34	0.52	0.65	0.20
Occidental	236	6	2.08	0.35	0.47	0.26
Occidental cultivars	212	6	1.98	0.37	0.46	0.20
Occidental landraces	24	4	1.99	0.21	0.42	0.48
Chinese cultivars + Occidental cultivars	419	9	2.64	0.45	0.57	0.16
Chinese cultivars + Occidental landraces	233	9	2.82	0.50	0.59	0.15
Chinese landraces + Occidental cultivars	358	13	2.80	0.43	0.59	0.27
Chinese landraces + Occidental landraces	170	10	3.35	0.49	0.65	0.25

With respect to the genetic diversity in the subgroups, 11 alleles were obtained within 353 Chinese accessions, and 5 alleles in 64 non-Chinese (Japanese and Korean) accessions. Observed heterozygosity was similar in both groups. With a further subdivision of the Chinese collection, a higher number of alleles was identified in the landraces (146 accessions) than in cultivars developed in breeding programs (207 accessions). Observed heterozygosis was similar in both groups (0.52 and 0.53 respectively) and lower than the expected, yielding a positive value of F (0.20 and 0.10 respectively).

In the Occidental group, four alleles were amplified in 24 Occidental landraces, and six alleles in 212 Occidental breeding cultivars. Observed heterozygosity was lower in the landraces compared with that in the breeding cultivars.

Similarly, the genetic variability in the analysis was considerably increased after combining Chinese landraces with Occidental breeding cultivars, while the effect of adding Occidental landraces to the Oriental cultivars was not significant. The number of heterozygous loci was higher using Chinese breeding cultivars, despite the lower heterozygosity of the Occidental landraces.

### Genetic relationship among the accessions

**Figure 2:**
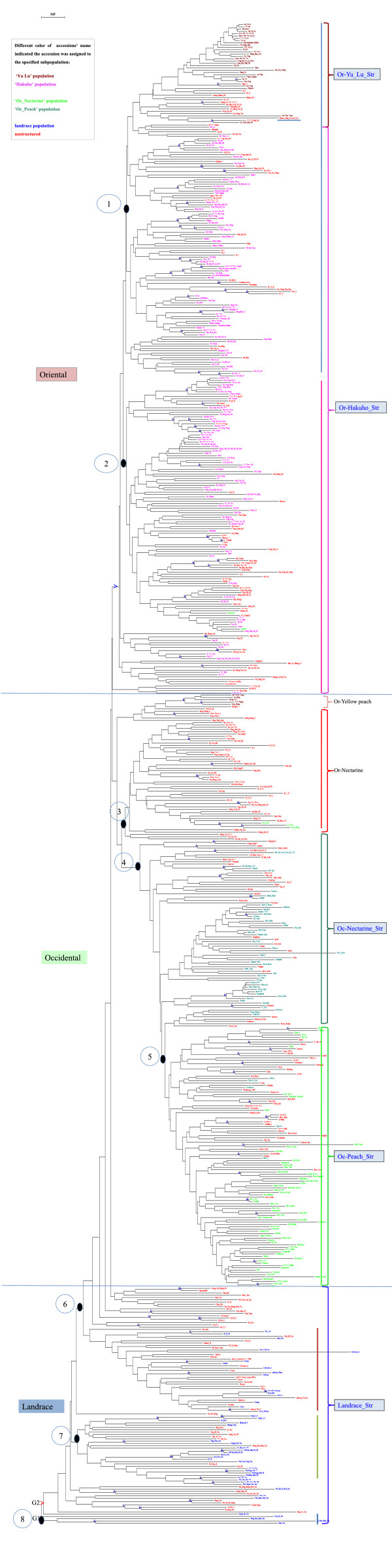
**Neighbour-joining tree for the 658 *prunus *accessions.** The tree was rooted using one wild relative species ‘Guang He Tao’ (*Prunus mira*. Koehne.) as outgroup. Bootstrap support values greater than 80% are shown in blue on the branches. Circled numbers beside the tree nodes indicate the 8 major groups. The colored parentheses indicate the clusters inferred by STRUCTURE analysis of 5 populations. The population ID are noted on the right. Accessions in different colors indicate they were assigned to corresponding populations. Unstructured accessions are in red.

A phylogenetic dendrogram (Figure 2, Additional file 1: Figure S1) based on genetic distances clearly divided the 658 accessions into two main groups: G1 and G2. Four wild peach related species fell into the G1 group as an outgroup, including the non-*persica* accessions ‘Gan Su Tao’ (*P*. *kansuensis*), ‘Hong Hua Shan Tao’ , ‘Bai Hua Shan Tao’ and ‘Shan Tao’ (*P*. *davidiana*), whilst ‘Guang He Tao’ (*P*. *mira* Koehne) clustered with the remaining peach accessions in G2. G2 contained all the *persica* accessions. The most genetically distinct accession was ‘Hong Ye Tao’. Seven major groups were clustered in G2, assigned 587 accessions, clustered not only according to the pedigree information and eco-geographical origin in the dendrogram, but also consistent with the structure-based membership assignment. The other accessions were clustered into several small groups. The founder cultivar ‘Chinese Cling’ , widely used in European and America breeding programs, clustered in the Oriental ‘Yu Lu’ group with accessions from the Zhejiang Province (at the top of the tree). Eleven yellow peaches and 52 nectarines from China clustered with the Occidental peach group. AMOVA and PCoA among 8 major groups based on the phylogenetic tree were analysed (Additional file 2: Table S2, Additional file 3: Table S3, Additional file 4: Figure S2). A variation of 75.90% was detected within individuals, while 19.76% variation was attributed in 8 clusters. The overall Fst in the 8 clusters was 0.19765 (p value < 0.05). A PCoA plot showed that the first and second coordinates accounted for 47.38% and 19.28% of the molecular variation. The first coordinate clearly separated Oriental accessions from Occidental accessions, while four wild related species and Chinese nectarines could not be separated from the two large Oriental clusters.

### Population structure

**Figure 3:**
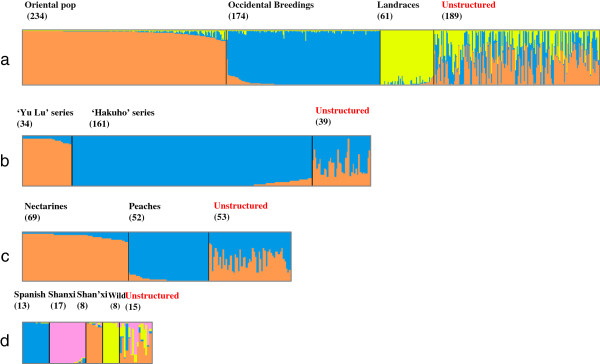
**Population stratification based on Bayesian clustering approaches for K = 1 to K = 10 (Each individual is shown as a thin vertical line, different subpopulations are separated by a black line and are in different colors). a** The first STRUCTURE step with 658 accessions, when K = 3. The subpopulation was displayed by DISTRUCT. Each subpopulation was ordered according to the membership coefficient. **b** Nested STRUCTURE analysis for the Oriental subpopulation which was further divided into two subpopulations: ‘Yu Lu’ and ‘Hakuho’. **c** Nested STRUCTURE analysis for the Occidental breeding subpopulation, mainly divided into ‘Nectarine’ and ‘Peach’ populations. **d** Nested STRUCTURE analysis for the landrace subpopulation. Note: Unstructured indicates the individuals which were not assigned to any subpopulation. The characters and numbers at the top of each population column give the name of each subpopulation and the number of individuals included.

According to the Evanno method [29], the collections were mainly divided into three subpopulations (K = 3). CLUMPP alignment of ten independent solutions for K = 3 gave pairwise ‘G’ values around 0.99, indicating that the assignment of accessions to the subpopulation was well correlated among runs. Considering the membership coefficient Q ≥ 80%, 469 accessions were clustered into three subpopulations (Figure 3a), one with Oriental breeding cultivars (234), one with Occidental breeding cultivars (174) and the third including both Oriental and Occidental landraces (61). The remaining accessions (189, unstructured) could not be assigned under the 80% membership coefficient criteria; almost 75% of them were Oriental and Occidental cultivars, and four of the founders used in the earlier USA breeding programs (‘Admiral Dewey’ , ‘Early Crawford’ , ‘Elberta’ , and ‘Chinese Cling’) also clustered within this admixed group.

The Oriental subpopulation of breeding cultivars was further divided into two groups (Figure 3b), one group including 34 ‘Yu Lu’ derived cultivars, of which 24 were from Zhejiang Province. Another group included 161 cultivars, of which 32 were Japanese cultivars, 59 were Chinese cultivars (all associated with one Japanese cultivar in their pedigree, principally the cultivars ‘Okubo’ , ‘Hakuho’ , ‘Sunago wase’), 22 were from the Shanxi collection and the remaining 49 cultivars from different geographic areas. Two founder cultivars, ‘Zao Shanghai Shui Mi’ (commonly called ‘Chinese Cling’ in Europe and America) and ‘Bai Hua Shui Mi’ and its offspring ‘Yu Hua Lu’ , were also assigned to this group.

The Occidental breeding subpopulation (with only the three Chinese cultivars ‘Ai Li Hong’ , ‘Ai Li Mi’ , and ‘Le Yuan’) further subdivided into two groups, one with 69 nectarines and two peaches (‘P 86 124’ , ‘Snow Flame’) and one with 52 peaches and only one nectarine (‘Silvery’) (Figure 3c).

The landraces subpopulation was further structured into four groups. The first included 13 Spanish landraces, the second included five wild related species, ancestral form peaches (‘Mao Tao’) and a low-chilling requirement old landrace (‘Nan Shan Tian Tao’), and the third group (Shan’xi subpopulation) included three ornamental cultivars (‘Shou Fen’ , ’01-42-45’ , ’03-5-16’) and five cultivars from Shanxi Province in the northwest of China. In the fourth group, Shanxi, there were nine very old landraces (‘Wu Yue Xian SX’ , ‘Ye Mao Tao SX’ , ‘Sx1-07’ , ‘Bai Lu Tao’ , ‘Qiu Fen Tao’ , ‘Yangqu Bai Tao’ , ‘Taigu Rou Tao’ , ‘Yuci Bai Tao’ , ‘Taiyuan Shui Mi_SX’) from Shanxi Province. This subpopulation also included eight other landraces (‘Nan Can Gong Tao’ , ‘Feicheng bai li 10’ , ‘Feicheng bai li 17’ , ‘Ge Gu Tao’ , ‘Mai Huang Pan Tao’ , ‘Shenzhou Shui Mi’ , ‘Taiyuan Shui Mi_ZZ’ and ‘Wu Yue Xian_ZZ’) from central China (Figure 3d).

**Figure 4:**
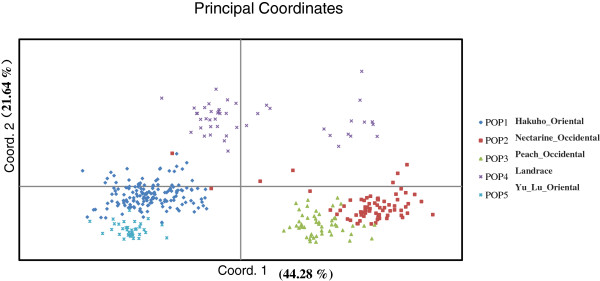
**Principal coordinate analysis (PCoA) of 469 *Prunus *accessions.** The different colors represent the five subpopulations inferred by nested STRUCTURE analysis. The first and second principal coordinates account for 47.38% and 19.28% of the total variation respectively.

Based on AMOVA analysis, most variation (68.64%) was detected within individuals, while less, but a significant part of the variation (27.24 6%) was attributed to variation among the five large subpopulations (Table 3). The overall Fst among the five subpopulations was 0.2723 (p value < 0.05). The pairwise Fst value in this study ranged from 0.20667 (between the ‘Hakuho’ and ‘Yu Lu’ subpopulations) to 0.44202 (between the Occidental ‘nectarine’ and ‘Yu Lu’ subpopulations). Pairwise Fst values between the two subpopulations within the Oriental and Occidental subpopulations were 0.20667 and 0.21877, respectively (Table 4). Genetic diversity of 469 structured accessions was also confirmed by PCoA (Figure 4). The first 3 axes together accounted for 79.55% of the variation. The first and second coordinates accounted for 44.48% and 21.64% of the molecular variation, with the first coordinate separating Oriental accessions from Occidental accessions, and the second coordinate the landraces from breeding cultivars.

**Table 3:** **Analysis of molecular variance (AMOVA) based on the 48 SSR loci of 469 *Prunus *accessions among inferred subpopulations by STRUCTURE analysis**

**Source of variation**	**d.f.**	**Sum of squares**	**Variance components**	**Fixation indices**	**Percentage of variation**
Among subpopulation	4	1420.993	1420.993	Fst = 0.2723	27.24
Among individuals within subpopulations	358	2674.449	0.40055 Vb	Fis = 0.2514	4.12
Within individuals	363	2421.000	6.66942 Vc	Fit = 0.3136	68.64
Total	725	6516.442	9.71653		

**Table 4:** Pairwise estimates of Fst based on 48 SSRs among the five subpopulations

	**‘Hakuho’_Or**	**Nectarine_Oc**	**Peach_Oc**	**Landrace**	**‘Yu_Lu’_Or**
‘Hakuho’_Or	0.0000				
Nectarine_Oc	0.31036	0.0000			
Peach_Oc	0.24248	0.21877	0.0000		
Landrace	0.20788	0.27049	0.27822	0.0000	
‘Yu_Lu’_Or	0.20667	0.44202	0.35046	0.35088	0.0000

Note: ‘Hakuho’_Or, Nectarine_Oc, Peach Oc, Landrace, ‘Yu_Lu’_Or indicate the subpopulations inferred by STRUCTURE analysis.

### Linkage disequilibrium

The extent of linkage disequilibrium (LD) was evaluated in the seven subpopulations with sample size larger than 20: Oriental, Occidental, Oriental ‘Yu Lu’ , Oriental ‘Hakuho’ , Occidental peaches, Occidental nectarines and, the landraces subpopulations.

A total of 3,148, 2,435 and 5,122 pairs of linked alleles were obtained in the three main subpopulations (Oriental, Occidental and landraces, respectively), within the same linkage group (intra-chromosome), 453 (14%) of the Oriental subpopulation, 333 (14%) of the Occidental and 805 (16%) of the landraces (Table 5). The percentage of intra-chromosome pair comparisons with significant LD (r^2^ > 0.1) was 17% in the Oriental subpopulation, 14% in the Occidental and 7% in the landraces subpopulation. In the Oriental and Occidental subpopulations, this numbers was considerably higher than that observed for inter-chromosome comparisons (6% and 4%, respectively), while landraces had the same proportion of inter and intra-chromosome comparisons in LD.

**Table 5:** Summary of LD parameters within 8 chromosomes in each subpopulation

**Subpopulation**	**Sample size**	**Number of comparisons**	**Inter-chromosome comparisons**	**% inter -chromosome r2 > 0.1**	**Intra-chromosome comparisons**	**% intra -chromosome r2 > 0.1**
Oriental	234	3,148	2,695	6%	453	17%
Hakuho	161	2,774	2,379	6%	395	20%
Yu Lu	34	1,851	1,566	36%	285	47%
Occidental	174	2,435	2,102	4%	333	14%
Nectarine	69	2,434	2,139	5%	295	17%
Peach	52	2,437	2,092	20%	345	29%
Landraces	61	5,122	4,317	7%	805	7%

The proportion of intra-chromosome comparisons in LD was higher in the nested than in the main subpopulations: 20% in Oriental-Hakuho, 47% in the Oriental-Yu Lu peaches, 17% in Occidental-nectarines and 29% in Occidental-peaches. The proportions of inter-chromosome comparisons in LD were 6%, 36%, 5% and 20%, respectively.

**Figure 5:**
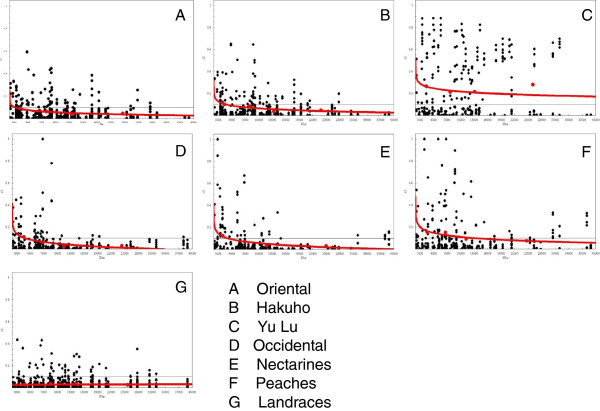
**LD decay plot in all subpopulations summarizing STRUCTURE and Nested STRUCTURE result.** The correlation between the X and Y axis indicates the decay trend of the LD coefficient (r^2^) with physical distance within intrachromosome At the top, **A**, **B** and **C** show the LD in the large ‘Oriental’ , ‘Hakuho’ and ‘Yu Lu’ subpopulations, respectively. In the middle, **D**, **E** and **F** show the LD in the large ‘Occidental’ , ‘Nectarine’ and ‘Peach’ subpopulations, respectively. **G** shows the LD level in the ‘landrace’ subpopulation. The horizontal line in each plot indicates r^2^ = 0.1.

The decay of LD with genetic map distance (Additional file 5: Figure S3) and physical distance (Figure 5) was calculated for each subpopulation. Figure 5 shows the LD variation with physical distance measured in Kbp. In the Oriental subpopulation, r^2^ reached the 0.1 threshold at 1,196 Kbp (3.36 cM) compared to 2,687 Kbp (5.50 cM) in the Occidental one. In the Chinese and European integrated subpopulation, few allele-comparisons were in LD (7%) and no tendency of decay with physical distance was observed. In the nested subpopulation, LD decayed faster in the Oriental-‘Hakuho’ subpopulation, reaching r^2^ = 0.1values at 2,757 Kbp (5.64 cM), compared to 3,153 Kbp (6.30 cM) for Occidental-nectarines, and 12,862 Kbp (24.93 cM) for Occidental-peaches. In the Oriental-‘Yu Lu’ subpopulation, the logarithmic curve representing the decay of LD with genetic distance intersected with the r^2^ = 1 value at 102.8 cM, out of the range of genetic distance of the peach chromosomes.

**Figure 6:**
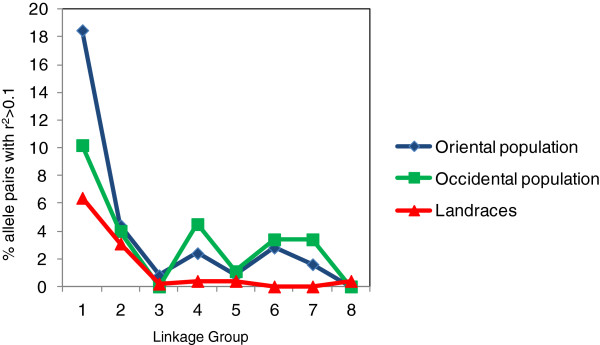
**Graph showing the percentage of alleles in LD in different linkage groups in three large subpopulations.** X-axis: each linkage group by number. Y-axis: the proportion of allele pairs with r^2^ > 0.1.

The LD level was also compared among eight different linkage groups (LG) within the three large Oriental, Occidental and landrace subpopulations, shown in Figure 6. In all subpopulations, the linkage group LG1 had the highest proportion of alleles in LD. Breeding subpopulations (Oriental and Occidental) had the greater proportion of pairs of alleles in LD in LG1, LG2, LG4 and LG7, while a low percentage of linked alleles was observed in LG3, LG5 and LG8. In landraces, linked alleles were observed mainly in LG1 and LG2, while the proportion on other linkage groups was practically negligible.

## Discussion

### SSR polymorphism and genetic diversity

Here we studied the variability of a heterogeneous collection of 658 peach genotypes. Close to two thirds of them were of Oriental origin (China, Japan and Korea) and the remainder from Occidental regions (Europe and USA). The sample included cultivars from both Oriental and Occidental breeding programs as well as landraces, wild peaches and other *Prunus* species closely related to peach. These accessions were analyzed with 48 SSRs in two different laboratories, the use of some common accessions as controls allowed the combination of the two datasets for the joint analysis of the data to identify and compare the variability intrinsic to each collection.

In total, the 48 polymorphic SSRs used amplified an average of 12.25 alleles per locus, 19% of them rare alleles, which is higher than the values observed in previous studies on genetic diversity in peach [5,16-20,28,30,31]. These high values can be explained by the large sample set and high heterogeneity of the sample.

The Oriental accessions contributed most to the variability of the sample, especially the landraces which, in general, are considered valuable in germplasm collections as sources of genetic diversity [5]. Some of the landraces studied here came from the north of China, especially the northwest, which is where peach originated. An average of 10 alleles per locus was amplified from 146 Chinese landraces. This is higher than the average of 6.4 previously reported in [28], where 104 landraces were analyzed with 53 SSRs. Some of the landraces have their own gene pools, especially those from Shanxi Province: ‘Bai Lu Tao’ ‘Qiu Fen Tao’ ‘Taigu Rou Tao’ ‘Yangqu Bai Tao’ ‘Jin Qiu’ ‘Wu Yue Xian’ and ‘Taiyuan Shui Mi’. Heterozygosity was lower in the Occidental than in the Oriental accessions, in part due to the limited genetic background in American and European peach breeding programs and also the low heterozygosity of the Occidental landraces caused by their self-propagation. This high proportion of heterozygous loci is consistent with [10]. After studying 45 peach cultivars and rootstocks from the USA, China, France, and Canada, these authors concluded that the highest levels of heterozygosity were detected in those cultivars from China. In summary, from our results, we deduce that it may be a desirable strategy to use Chinese germplasm to increase genetic diversity in Occidental cultivars, while any introgression of new alleles should be carried out without disrupting existing allele combinations associated with superior traits bred into these cultivars.

### Phylogenetic clusters

Phylogenetic analysis grouped all accessions into 8 major clusters consistent with geographic origin, domestication history and mating system. The result was also agreement with PCoA and population structure analysis: (1) Oriental accessions were separated from Occidental accessions; (2) breeding cultivars were separated from old landraces; (3) the Oriental-Yu Lu group was separated from the Hakuho group; the Occidental-peach group was separated from the nectarine group.

Collections from Japan and China clustered together and could not be separated clearly, revealing similar origin and genetic background as well as the effect of breeding strategies at the whole genome level [4]. This effect has also been reported in Occidental breeding material [32]. ‘Chinese Cling’ , used as founder in western countries, where it is known as ‘Shanghai Shui Mi’ , grouped with ‘Qi Yuan Shui Mi’ and ‘Ren Pu Shui Mi’ which originated from Zhejiang Province. While another founder cultivar ‘Zao Shanghai Shui Mi’ (called ‘Chinese Cling’ in Japan) clustered within Japanese peaches. The relatively long genetic distance between ‘Chinese Cling’ and ‘Zao Shanghai Shui Mi’ is probably because ‘Shanghai Shui Mi’ was not a single but a group of cultivars [4]. Two Spanish landraces, the white non-melting peach ‘Binaced’ and flat white peach ‘Paraguayo Delfin’ , clustered with Chinese peaches. These two cultivars have been studied by different Spanish research groups [15,17,20] and always kept separate from other Spanish materials in breeding. Here the results provide important clues that these two cultivars were probably selected from China or obtained from seed or clonal propagation of a Chinese cultivar. Since the Occidental nectarine cultivars ‘Mayfire’ and ‘NJN76’ were widely used as parents in Chinese breeding programs, 52 Chinese nectarines clustered within the Occidental group [5].

Two large Oriental groups (‘Yu Lu’ and ‘Hakuho’) can be explained by their pedigree relationship. Mutation and seedling selection was the dominant way to select new cultivars in the ‘Yu Lu’ subpopulation. Selfing and crossing with ideal parent materials were adopted in the ‘Hakuho’ subpopulation.

Three occidental cultivars, ‘Dixon_SX’ , ‘Fantasia_Or’ and ‘Flavortop_Or’ , from Shanxi germplasm did not fit in the Occidental group. ‘Hong Bao Shi’ might have the same identity as ‘Red Diamond’ with a genetic distance less than 0.05. This information will be useful in germplasm collections to efficiently preserve peach cultivars by identifying and removing redundancies, focusing resources on poorly represented groups or validating the new selected cultivars.

### Population structure

Here we applied a nested clustering strategy with the STRUCTURE software. This method has been previously used with large sample sizes, exhibiting a strong capability to assign individuals into populations [33-36]. For example, 566 South-American *Solanum* section Petota produced an optimal partitioning into 44 groups with this two-step method [34]. In *Pisum*, three subpopulations, corresponding approximately to landrace, cultivar and wild *Pisum*, were obtained in the first STRUCTURE step, and 14 sub-subpopulations were obtained through the second STRUCTURE step, which correlated with the taxonomic sub-division of *Pisum* according to phenotypic traits and/or geographical origin [35]. Likewise, a deep division has been observed in Northeast Spanish apple accessions using this method, identifying two robust sub-groups [36]. In our study, a first run of STRUCTURE distinguished three subpopulations according to geographical location and domestication history, while 189 were not assigned to a subpopulation. In this first step, all landraces (Spanish and Chinese) clustered together while a further analysis separated Chinese from non-Chinese landraces. This may indicate that Spanish landraces are from one or a few common ancestors. Previous research on the evolutionary history of peach has also indicated a high probability that the Spanish non-melting peaches evolved from northwest Chinese peaches [4].

Still in the first step, a clear divergence between Oriental and Occidental commercial subpopulations reveals the existence of different breeding sources of germplasm in Chinese and western countries. In the Chinese breeding program, cultivars from Japan, especially ‘Hakuho’ , ‘Hakuto’ and ‘Okubo’ , have had a great impact on Chinese peach breeding. All are selected from ‘Chinese Cling’ (‘Shanghai Shui Mi’). After being introduced into China, ‘Hakuho’ was mainly grown in the south of China as a good quality, soft-melting honey peach, and ‘Okubo’ was used in the north as a parent for both peach and nectarine selections. Because of the kinship between ‘Hakuho’ and ‘Okubo’ (both from ‘Hakuto’), it is not possible to distinguish the breeding cultivars by where they are grown. In Occidental breeding programs, a few accessions were intensively used as founders in early breeding programs, producing a dramatic reduction of variability. One of the founders reported in the literature is ‘Chinese Cling’ , however next-generation sequencing of this cultivar have revealed the low prevalence of its genome in current western commercial cultivars [21].

### Linkage disequilibrium

Linkage disequilibrium (LD) has been especially used for marker-trait association in whole genome studies in plants. Knowing the level of LD is crucial in the design of association mapping studies. We found that LD decays approximately 43% faster in Oriental (3.85 cM) than in Occidental subpopulations (5.50 cM), while no decay of LD with genetic distance was observed in the landraces subpopulation. These three subpopulations are composed of nested subpopulations, which could lead to miscalculation of the LD. LD analysis in the nested subpopulations showed high LD extensions in the Oriental-Yu Lu and Occidental-peaches subpopulations. In both, the proportion of inter-chromosome pairwise allele comparisons of LD was much higher than that observed in the other subpopulations (20-36% compared to 4-7%). The large LD in the Yu Lu subpopulation is due to the intense pedigree relationship: most originated from the Fenghua Honey Peach Institute and 18 out of the 34 were considered to be mutants or seedlings selected from Yu Lu. The larger LD extension in Occidental peaches seems to be the result of the remaining subpopulation stratification in the LD estimation. In the Occidental subpopulations (Nectarines and Peaches), LD decayed at 6.3 cM and 24.9 cM, respectively, contrasting with the 13–15 cM previously reported in [15], using practically the same set of Occidental accessions. These discrepancies could be due to the inclusion of a few Chinese accessions in our subpopulation but also to the remaining population stratification.

LD intensity in the ‘landrace’ subpopulation was low and we did not observe decay with genetic distance. A similar report on 104 Chinese landraces also found low levels of LD in northwest China and middle and lower reaches of the Changjiang River subpopulations [28]. Extremely low levels of LD in landrace and wild accessions have also been observed in other species, such as wild French grape (2.7 cM compared to 16.8 cM for cultivated French grape) [25,26] and maize (1 kb in landraces, 2 kb in diverse inbred lines and 100 kb in commercial elite inbred lines) [24]. A rapid decline of LD was observed in a wild, strictly self-incompatible, cherry subpopulation compared to a cultivated sweet cherry [37]. The absence of LD, which may suggest that no “phylogeny” of accessions exists, has been found in 76 Arabidopsis thaliana lines tested with 163 SNPs [38]. The high genotypic variation, low LD and phenotypic variation indicate that, with more markers, landrace subpopulations could be an ideal group for further association mapping.

These results demonstrate that peach germplasm is potentially a valuable resource for association genetics. The accessions obtained in breeding programs come from a limited number of progenitors and, consequently, have a reduced level of variability. This means that the genetic variants responsible for the observed phenotypic traits are reduced and fixed, trimming down the presence of minor-effect alleles which are difficult to detect through association mapping. If new sources of alleles are needed, a population of landraces is available.

The level of LD in different genome regions is variable because of the selection and recombination rate. The significant difference in LD among different linkage groups provides more information to determine the marker density needed in association studies [38]. The knowledge of LD extension in, for example, linkage group 4, will be quite useful when choosing SSR or SNP markers to identify candidate genes and QTLs responsible for the synthesis of linalool and lactone, which contribute to the peach and nectarine volatile [39,40]. Flesh texture (melting/non-melting) correlated with the endo-polygalacturonase gene could be another interesting trait in this linkage group [41]. The recent publication on the peach genome [21] will be of great help to explore diversity on the whole-genome scale.

## Conclusions

By jointly analysing Occidental (European and North-American) and Chinese genotypic data, we were able to estimate the effect of using Chinese germplasm in Occidental breeding programs and vice versa. We demonstrate that Occidental elite lines could be a source of variability in Chinese breeding programs, but, as Chinese landraces have higher levels of genetic variability, they would have a greater effect on increasing genetic diversity when used in breeding programs in western countries. The unambiguous distinction between Oriental and Occidental subpopulations indicates that quite different genetic backgrounds were used as breeding materials in China, Japan, Europe and America. In general, LD decays faster in Oriental germplasm, so a higher density of markers should be used in association mapping, however previous knowledge of the LD in the population study will be always required. Landraces have low levels of LD, making them a good tool for fine mapping of traits through LD mapping.

## Methods

### Plant material and DNA extraction

A total of 434 *Prunus* accessions, 429 *Prunus persica* (L.) Batsch and five peach related species (one *P. mira,* three *P. davidiana* and one *P. kansuensis*) were chosen. The accessions studied were highly heterogeneous, covering different geographic regions (split as Oriental and Occidental) as well as different domestication history (wild, landraces and breeding). Among them, 353 accessions originated from China, 64 originated from Japan and Korea, including breeding cultivars and landraces, and twelve accessions were introduced from west countries. Most accessions were collected from five germplasm collections: the National Peach Germplasm repository at Jiangsu Academy of Agriculture Sciences (Southeast Region), Fenghua Honey Peach Research Institute (Southeast Region), Shanxi Academy of Agriculture Sciences (Central Region), Southwest University (Southwest Region) and the Zhengzhou Fruit Research Institute (Chinese Academy of Agriculture Sciences, Central Region). Some old local cultivars were obtained from the northwest of China as described in Additional file 6: Table S1.

Young terminal leaves were collected and frozen in liquid nitrogen. Total genomic DNA was extracted from 1 g frozen leaf tissue with a modified CTAB procedure, and the concentration quantified by ultraviolet spectrophotometer (Beckman Coulter DU800), then diluted to 20 ng/μl for PCR amplification.

### SSR amplification

The 434 Oriental accessions and six additional Occidental accessions were amplified with 48 SSR primer pairs (Table 6), 45 previously used by Aranzana [15] and PTS1-SSR, EPPCU1775 and PceGA25, also highly polymorphic. All forward primers were fluorescently labelled and PCR amplified using an Eppendorf Mastercycler 5333/5331 thermocycler 114 (Gradient No. 5331–41264, Germany), with amplification reactions and temperature cycles according to the protocol used by [18]. Fluorescently labelled PCR fragments were separated by capillary electrophoresis in an ABI PRISM 3130 DNA Analyser (Applied Biosystems, Foster City, CA, USA).

**Table 6:** Characteristics of the SSRs used in the present study

**Locus**	**Ta**^**1 **^**(°C)**	**Linkage group**^**2**^	**Genetic position (cM)**^**3**^	**Physical position**^**4**^	**Fluorescent labelling**^**5**^	**References**
UDP96-018	57	1	1	scaffold_1:1299253	FAM	Cipriani et al., 1999 [42]
CPPCT027	55	1	23.1	scaffold_1:12409317	NED	Aranzana et al., 2002 [32]
UDP96-005	57	1	29.2	scaffold_1:13903361	FAM	Cipriani et al., 1999 [42]
EPDCU1090	57	1	32	scaffold_1:22653855	HEX	Howad et al., 2005 [43]
CPPCT026	55	1	33.9	scaffold_1:31792505	FAM	Aranzana et al., 2002a [32]
Pchgms3	60	1	37.5	scaffold_1:27692065	VIC	Sosinski et al., 2000 [44]
BPPCT020	57	1	52.6	scaffold_1:33281268	HEX	Dirlewanger et al., 2002 [30]
CPPCT042	57	1	62.5	scaffold_1:39307938	HEX	Aranzana et al., 2002a [32]
CPPCT029	55	1	65.1	scaffold_1:40195426	HEX	Aranzana et al., 2002a [32]
CPPCT044	58	2	7.2	scaffold_2:10280697	HEX	Howad et al., 2005 [43]
UDP98-025	57	2	9.6	scaffold_2:10872102	HEX	Cipriani et al., 1999 [42]
BPPCT001	57	2	20.9	scaffold_2:16134154	HEX	Dirlewanger et al., 2002 [30]
UDP96-013	57	2	27.8	scaffold_2:18895940	FAM	Cipriani et al., 1999 [42]
pchgms1	55	2	35.1	scaffold_2:21255607	FAM	Sosinski et al., 2000 [44]
BPPCT024	57	2	36.3	scaffold_2:22674207	HEX	Dirlewanger et al., 2002 [30]
PceGA34	50	2	43.9	scaffold_2:25199147	FAM	Downey and Iezzoni., 2000 [45]
BPPCT007	57	3	11.2	scaffold_3:2741939	HEX	Dirlewanger et al., 2002 [30]
BPPCT039	57	3	18	scaffold_3:5802709	NED	Dirlewanger et al., 2002 [30]
CPPCT002	52	3	31.9	scaffold_3:16205250	NED	Aranzana et al., 2002a [32]
UDP96-008	57	3	36.4	scaffold_3:16946762	FAM	Cipriani et al., 1999 [42]
PTS1-SSR	53	4	5.2	scaffold_4:1414471	HEX	Illa et al., 2011 [37]
UDP98-024	57	4	11.3	scaffold_4:3499623	FAM	Testolin et al., 2000 [31]
CPPCT005	52	4	10.4	scaffold_4:10269880	FAM	Aranzana et al., 2002a [32]
UDP96-003	57	4	28.3	scaffold_4:8757450	FAM	Cipriani et al., 1999 [42]
BPPCT015	57	4	44	scaffold_4:12546880	NED	Dirlewanger et al., 2002 [30]
CPPCT046	58	4	45.4	scaffold_4:14476745	HEX	Aranzana et al., 2002a [32]
EPPCU1775	57	4	52	scaffold_4:22684553	HEX	Howad et al., 2005 [43]
CPPCT040	57	5	1.5	scaffold_5:993617	HEX	Aranzana et al., 2002a [32]
UDP97-401	57	5	11	scaffold_5:5940392	HEX	Cipriani et al., 1999 [42]
BPPCT017	57	5	20.1	scaffold_5:11174442	HEX	Dirlewanger et al., 2002 [30]
BPPCT037	57	5	25.6	scaffold_5:12312049	FAM	Dirlewanger et al., 2002 [30]
BPPCT038	57	5	32.9	scaffold_5:14658198	NED	Dirlewanger et al., 2002 [30]
PCeGA25	58	5	28.4	scaffold_5:12835942	FAM	Downey and Iezzoni., 2000 [45]
CPPCT013	59	5	29.2	scaffold_5:12835904	FAM	Aranzana et al., 2002a [32]
CPSCT006	62	5	21.7	scaffold_5:11533644	FAM	Mnejja et al., 2004 [46]
BPPCT014	57	5	44	scaffold_5:16626108	FAM	Dirlewanger et al., 2002 [30]
UDP96-001	57	6	17.5	scaffold_6:7040757	VIC	Cipriani et al., 1999 [42]
CPPCT015	50	6	35.8	scaffold_6:16352480	PET	Aranzana et al., 2002a [32]
pchcms5	57	6	44.7	scaffold_6:19166654	FAM	Sosinski et al., 2000 [44]
BPPCT025	57	6	56.4	scaffold_6:21129947	FAM	Dirlewanger et al., 2002 [30]
CPPCT030	50	6	80.2	scaffold_6:26851012	FAM	Aranzana et al., 2002a [32]
CPPCT022	50	7	18.6	scaffold_7:10225365	FAM	Aranzana et al., 2002a [32]
pchgms6	58	7	19.4	scaffold_7:10439493	HEX	Aranzana et al., 2002a [32]
CPPCT033	50	7	38.9	scaffold_7:16702195	FAM	Aranzana et al., 2002a [32]
PMS02	55	7	47.8	scaffold_7:18106236	PET	Cantini et al., 2001 [47]
BPPCT006	57	8	14.1	scaffold_8:5982783	FAM	Dirlewanger et al., 2002 [30]
CPPCT006	59	8	24.8	scaffold_8:13659021	FAM	Aranzana et al., 2002a [30]
UDP98-409	57	8	44.5	scaffold_8:17783528	FAM	Cipriani et al., 1999 [42]

Note: ^1^: indicates annealing temperatures.

^2^: indicates linkage group information.

^3^: position of the SSR markers in the T × E linkage map.

^4^: physical position of the SSR markers in the peach genome sequence v1.0 ( http://www.rosaceae.org/peach/genome).

^5^: fluorescent labeled dye added to the forward primer.

### Data collection

#### Analysis of genetic diversity and population structure

The genotypic data obtained was added to the Occidental data matrix of 236 accessions previously genotyped at IRTA (Institut de Recerca i Tecnologia Agroalimentàries), using the data of six accessions as size control. The following parameters of variability were calculated with PowerMarker 3.25 [48]: number of observed alleles per locus (Ao), effective number of alleles (Ae), observed heterozygosity (Ho), expected heterozygosity (He), Wright’s fixation index (f = 1-Ho/He) and power of discrimination for each locus (PD). These parameters were calculated for the whole sample and for small subsets, considering their geographic origin or breeding status. Based on the Nei and Li [49] genetic distance estimation method, a Neighbour-joining dendrogram (with 1,000 bootstraps) was constructed with TREECON 1.3b [50]. The tree was rooted using one wild related species ‘Guang He Tao’ (*Prunus mira.* Koehne.) as outgroup.

### Number of alleles inference in different subsets

Rarefaction curves were drawn to compare variability in the different sample subsets of different sample size. For these, random groups of accessions of sample size from 2 to 657 were selected by bootstrap and the mean value of Ao and Chebyshev 95% confidence intervals were calculated.

### Population structure

Data from 25 SSR markers were selected to study population structure using STRUCTURE v.2.0 software [51], adopting an admixture model and correlated alleles, with burn-in and MCMC 100,000 and 1,000,000 cycles respectively. Each locus used in STRUCTURE analysis was separated from one another by at least 15 cM. K values were set from one to ten with assumption populations, running ten independent repeats per K. The most likely number of subpopulations was calculated according to Evanno’s method [29]. The average membership coefficient for each accession was calculated using CLUMPP [52,53]. Accessions were assigned to a subpopulation when the membership coefficients were Q ≥0.8. Results were plotted with DISTRUCT software [54]. A second level analysis (nested) of population structure with the same software and parameters was carried out on each of the subpopulations detected in the first STRUCTURE run.

### Analysis of molecular variance (AMOVA)

The genetic variation within and among subpopulations of 469 *Prunus* accessions and pairwise Fst were measured by analysis of molecular variance (AMOVA) using Arlequin v3.5 software. The threshold for statistical significance was determined by running 1000 permutations [55]. Principal coordinate analysis of all inferred subpopulations based on genetic distance matrix was also carried out, using the GenAlEx 6.5 software [56].

### Analysis of linkage disequilibrium

Linkage disequilibrium (LD) was calculated in all subpopulations obtained in the STRUCTURE analysis, including the three large subpopulations from the first structure step as well as the four small subpopulations obtained by nested STRUCTURE analysis. Alleles with frequencies lower than 5% were removed in case of rare alleles causing an effect of inflation on LD estimation and on P-value. The correlation coefficient r^2^ between each pair of alleles among 48 loci was calculated with pairwise LD analysis in PowerMarker 3.25 software, considering unphased genotype data [48]. The exact test was implemented to identify whether the two loci were significantly correlated, using the method set as permutation with a convergence bound of 0.05.

For each subpopulation, intra-chromosome LD was plotted against distance (measured in cM and Kbp). The curve of the variation of r^2^ values with physical distance was calculated using the average values for each of four subsets of an equal number of pair comparisons covering adjacent intervals over genetic distance. The threshold r^2^ = 0.1 was used for decay distance estimation as suggested by [57].

## Competing interests

The authors declare that they have no competing interests.

## Authors’ contributions

XWL and XQM performed the experiment, XWL and MJA analyzed the statistical data and drafted the manuscript. HJJ, MLY, RJM, LRW, ZJS, KC, LN, JBT, MJC and MX provided the plant material and phenotype information. ZSG, PA, MLY, LRW initiated the project and designed the research framework. ZSG and PA reviewed the manuscript. All authors read and approved the final manuscript.

## Supplementary Material

Additional file 1: Figure S1 Neighbour-joining tree with bootstrap support values (>80%, based on 1,000 bootstraps) for the 658 *Prunus* accessions. The tree was rooted using one wild relative species Guang He Tao (*Prunus mira*. Koehne.) as outgroup. The colored parentheses indicate the clusters which were inferred by STRUCTURE analysis of 5 subpopulations, the subpopulation IDs are noted on the right. Accessions with different colors indicate they were assigned to corresponding subpopulations. Unstructured accessions are in red. Click here for file

Additional file 2: Table S2 Analysis of molecular variance (AMOVA) based on the 48 SSR loci of 587 *Prunus* accessions among 8 major groups by phylogenetic analysis (p < 0.05). Click here for file

Additional file 3: Table S3 Pairwise estimates of Fst based on 48 SSRs among 8 major groups (including 587 accessions) (p < 0.05). Click here for file

Additional file 4: Figure S2 Principal coordinate analysis (PCoA) of 587 *prunus* accessions. The different colors represent the 8 major groups inferred by phylogenetic analysis. The first and second principal coordinates account for 44.28% and 21.64% of the total variation, respectively. Click here for file

Additional file 5: Figure S3 LD decay plot in the subpopulations summarizing STRUCTURE and Nested STRUCTURE result. The correlation between the X and Y axis indicates the decay trend of the LD coefficient (r^2^) with genetic distance within intrachromosome. At the top, A, B and C show the LD level in the large ‘Oriental’ , ‘Hakuho’ and ‘Yu Lu’ subpopulations, respectively. In the middle, D, E and F show the LD level in the large ‘Occidental’ , ‘Nectarine’ and ‘Peach’ subpopulations, respectively. G shows the LD level in the ‘Landrace’ subpopulation. The horizontal line in each plot indicates r^2^ = 0.1. Click here for file

Additional file 6: Table S1 Detailed information on the studied cultivars including main fruit traits, pedigree, origin and geographic area in China. Click here for file

## References

[B1] Potter D Eriksson T Evans RC Oh S Smedmark JEE Morgan DR Kerr M Robertson KR Arsenault M Dickinson TA Campbell CS Phylogeny and classification of rosaceae Plant Syst Evol 2007 266 1–2 5 43

[B2] Strada GD Fideghelli C Le cultivar de drupacee introdottee del 1991 al 2001 L’ Informatore Agrario 2003 59 41 65 7024052888

[B3] Bielenberg D Gasic K Chaparro JX An Introduction to Peach (Prunus Persica) Genetics and Genomics of Rosaceae 2009 New York: Springer 223 234

[B4] Wang ZH Zhuang EJ China Fruit Monograph-Peach Flora 2001 Beijing: China Forestry Press

[B5] Zhang AM Yoon JH Liu DC Song WS Liu WS Li SH Genetic diversity and ecogeographical phylogenetic relationships among peach and nectarine cultivars based on simple sequence repeat (SSR) markers J Am Soc Hortic Sci 2006 131 4 513 521

[B6] Xu DH Wahyuni S Sato Y Yamaguchi M Tsunematsu H Ban T Genetic diversity and relationships of japanese peach (*Prunus persica* L.) cultivars revealed by AFLP and pedigree tracing Genet. Resour. Crop Evol 2006 53 5 883 889 10.1007/s10722-004-0575-z

[B7] Yamamoto T Mochida K Hayashi T Shanhai suimitsuto, one of the origins of japanese peach cultivars J Jpn Soc Hortic Sci 2003 72 2 116 121 10.2503/jjshs.72.116

[B8] Aranzana MJ Illa E Howad W Arús P A first insight into peach *[prunus persica* (L.) batsch] SNP variability Tree Genet Genomes 2012 8 6 1359 1369 10.1007/s11295-012-0523-6

[B9] Scorza R Mehlenbacher SA Lightner GW Inbreeding and co-ancestry of freestone peach cultivars of the eastern United States and implications for peach germplasm improvement J Am Soc Hortic Sci 1985 110 547 552

[B10] Martínez-Gómez P Arulsekar S Potter D Gradziel TM An extended interspecific gene pool available to peach and almond breeding as characterized using simple sequence repeat (SSR) markers Euphytica 2003 131 3 313 322 10.1023/A:1024028518263

[B11] Hancock JF Scorza R Lobos GA Hancock JF Peaches Temperate fruit crop breeding 2008 New York: Springer 265 298

[B12] Sansavini S Gamberini A Bassi D Peach breeding, genetics and new cultivar trends Acta Horticulturae 2006 713 23 48

[B13] Baird WV Ballard RE Rajapakse S Abbott AG Progress in *prunus* mapping and application of molecular markers to germplasm improvement HortSci 1996 31 1099 1106

[B14] Infante R Martínez Gómez P Predieri S Quality oriented fruit breeding: peach [*Prunus persica* (L.) batsch] J Food Agriculture & Environ 2008 6 2 342 356

[B15] Aranzana MJ Abbassi EK Howad W Arus P Genetic variation, population structure and linkage disequilibrium in peach commercial varieties BMC Genet 2010 11 69 20646280 10.1186/1471-2156-11-69 PMC2915947

[B16] Aranzana MJ Carbó J Arús P Microsatellite variability in peach [*Prunus persica* (L.) batsch]:cultivar identification, marker mutation, pedigree inferences and population structure Theor Appl Genet 2003 106 8 1341 1352 12750778 10.1007/s00122-002-1128-5

[B17] Bouhadida M Moreno MÁ Gonzalo MJ Alonso JM Gogorcena Y Genetic variability of introduced and local Spanish peach cultivars determined by SSR markers Tree Genet Genomes 2010 7 2 257 270

[B18] Xie R Li X Chai M Song L Jia H Wu D Chen M Chen K Aranzana MJ Gao Z Evaluation of the genetic diversity of Asian peach accessions using a selected set of SSR markers Sci Hortic 2010 125 4 622 629 10.1016/j.scienta.2010.05.015

[B19] Cheng Z Huang H SSR fingerprinting Chinese peach cultivars and landraces (*Prunus persica*) and analysis of their genetic relationships Sci Hortic 2009 120 2 188 193 10.1016/j.scienta.2008.10.008

[B20] Wünsch A Carrera M Hormaza JI Molecular characterization of local Spanish peach [*Prunus persica* (L.) batsch] germplasm Genet Resour Crop Evol 2005 53 5 925 932

[B21] Verde I Abbott AG Scalabrin S Jung S Shu SQ Marroni F Zhebentyayeva T Dettori MT Grimwood BJ Cattonaro F Zuccolo A Rossini L Jenkins J Vendramin E Meisel LA Decroocq V Sosinski B Prochnik S Mitros T Policriti A Cipriani G Dondini L Ficklin S Goodstein DM Xuan P Fabbro CD Aramini V Copetti D Gonzalez S Horner DS The International Peach Genome Initiative The high-quality draft genome of peach (*Prunus persica*) identifies unique patterns of genetic diversity, domestication and genome evolution Nat Genet 2013 45 487 494 10.1038/ng.2586 23525075

[B22] Gupta PK Rustgi S Kulwal PL Linkage disequilibrium and association studies in higher plants: present status and future *prospects* Plant Mol Biol 2005 57 4 461 485 10.1007/s11103-005-0257-z 15821975

[B23] Abdurakhmonov IY Abdusattor A Application of association mapping to understanding the genetic diversity of plant germplasm resources Int J Plant Genomics 2008 2008 574927 18551188 10.1155/2008/574927 PMC2423417

[B24] Sorkheh K Malysheva-Otto LV Wirthensohn MG Tarkesh-Esfahani S Martínez-Gómez P Linkage disequilibrium, genetic association mapping and gene localization in crop plants Genet Mol Biol 2008 31 4 805 814 10.1590/S1415-47572008005000005

[B25] Barnaud A Lacombe T Doligez A Linkage disequilibrium in cultivated grapevine, vitis vinifera L Theor Appl Genet 2006 112 4 708 716 10.1007/s00122-005-0174-1 16402190

[B26] Barnaud A Laucou V This P Lacombe T Doligez A Linkage disequilibrium in wild french grapevine,*Vitis vinifera* L. Subsp silvestris Heredity 2010 104 5 431 437 10.1038/hdy.2009.143 19844269

[B27] Jannoo N Grivet L Dookun A D’Hont A Glaszmann JC Linkage disequilibrium among modern sugarcane cultivars Theor Appl Genet 1999 99 6 1053 1060 10.1007/s001220051414

[B28] Cao K Wang L Zhu G Fang W Chen C Luo J Genetic diversity, linkage disequilibrium, and association mapping analyses of peach (*Prunus persica*) landraces in China Tree Genet Genomes 2012 8 5 975 990 10.1007/s11295-012-0477-8

[B29] Evanno G Regnaut S Goudet J Detecting the number of clusters of individuals using the software STRUCTURE: a simulation study Mol Ecol 2005 4 8 2611 2620 15969739 10.1111/j.1365-294X.2005.02553.x

[B30] Dirlewanger E Cosson P Tavaud M Aranzana MJ Poizat C Zanetto A Arús P Laigret F Development of microsatellite markers in peach [*prunus persica* (L.) batsch] and their use in genetic diversity analysis in peach and sweet cherry (*prunus avium* L.) Theor Appl Genet 2002 105 1 127 138 10.1007/s00122-002-0867-7 12582570

[B31] Testolin R Marrazzo T Cipriani G Quarta R Verde I Dettori MT Pancaldi M Sansavini S Microsatellite DNA in peach (*Prunus persica* L. Batsch) and its use in fingerprinting and testing the genetic origin of cultivars Genome 2000 43 3 512 520 10902716

[B32] Aranzana MJ Garcia-mas J Carbó J Arús P Development and variability analysis of microsatellite markers in peach Plant Breed 2002 121 1 87 92 10.1046/j.1439-0523.2002.00656.x

[B33] Wang S Lewis JCM Jakobsson M Ramachandran S Ray N Bedoya G Rojas W Parra MV Molina JA Gallo C Poletti G Hill K Hurtado AM Labuda D Klitz W Barrantes R Bortolini MC Salzano FM Petzl-Erler ML Tsuneto LT Llop E Rothhammer F Excoffier L Feldman MW Rosenberg NA Ruiz-Linares A Genetic variation and population structure in native americans PLoS Genet 2007 3 11 185 10.1371/journal.pgen.0030185 PMC2082466 18039031

[B34] Jacobs MM Smulders MJ van den Berg RG Vosman B What’s In a name; genetic structure in solanum section petota studied using population-genetic tools BMC Evol Biol 2011 11 1 42 10.1186/1471-2148-11-42 21310063 PMC3045909

[B35] Jing R Vershinin A Grzebyta J Shaw P Smykal P Marshall D Ambrose MJ Ellis TH Flavell AJ The genetic diversity and evolution of field pea (*Pisum*) studied by high throughput retrotransposon based insertion polymorphism (RBIP) marker analysis BMC Evol Biol 2010 10 1 44 10.1186/1471-2148-10-44 20156342 PMC2834689

[B36] Urrestarazu J Miranda C Santesteban LG Royo JB Genetic diversity and structure of local apple cultivars from northeastern spain assessed by microsatellite markers Tree Genet Genomes 2012 8 6 1163 1180 10.1007/s11295-012-0502-y

[B37] Arunyawat U Capdeville G Decroocq V Mariette S Linkage disequilibrium in french wild cherry germplasm and worldwide sweet cherry germplasm Tree Genet Genomes 2012 8 4 737 755 10.1007/s11295-011-0460-9

[B38] Nordborg M Borevitz JO Bergelson J Berry CC Chory J Hagenblad J Kreitman M Maloof JN Noyes T Oefner PJ Stahl EA Weigel D The extent of linkage disequilibrium in *Arabidopsis thaliana* Nat Genet 2002 30 2 190 193 10.1038/ng813 11780140

[B39] Illa E Eduardo I Audergon JM Barale F Dirlewanger E Li X Moing A Lambert P Dantec L Gao Z Poëssel J Pozzi C Rossini L Vecchietti A Arús P Howad W Saturating the *Prunus* (stone fruits) genome with candidate genes for fruit quality Mol Breed 2010 28 4 667 682

[B40] Eduardo I Chietera G Pirona R Pacheco I Troggio M Banchi E Bassi D Rossini L Vecchietti A Pozzi C Genetic dissection of aroma volatile compounds from the essential oil of peach fruit: QTL analysis and identification of candidate genes using dense SNP maps Tree Genet Genomes 2013 9 1 189 204 10.1007/s11295-012-0546-z

[B41] Peace CP Crisosto CH Gradziel TM Endopolygalacturonase: a candidate gene for *freestone* and *melting flesh* in peach Mol Breed 2005 16 1 21 31 10.1007/s11032-005-0828-3

[B42] Cipriani G Lot G Huang WG Marrazzo MT Peterlunger E Testolin R AC/GT and AG/CT microsatellite repeats in peach [*prunus persica* (L.) batsch]: isolation, characterisation and cross-species amplification in *prunus* Theor Appl Genet 1999 99 1–2 65 72

[B43] Howad W Yamamoto T Dirlewanger E Testolin R Cosson P Cipriani G Monforte AJ Georgi L Abbott AG Arus P Mapping with a few plants: using selective mapping for microsatellite saturation of the *Prunus* reference map Genetics 2005 171 3 1305 1309 10.1534/genetics.105.043661 16118196 PMC1456826

[B44] Sosinski B Gannavarapu M Hager LD Beck LE King GJ RyderCD RS Baird WV Ballard RE Abbott AG Characterization of microsatellite markers in peach [*prunus persic*a(L.) batsch] Theor Appl Genet 2000 101 421 10.1007/s001220051499

[B45] Downey SL Iezzoni AF Polymorphic DNA markers in black cherry (*Prunus serotina*) are identified using sequences from sweet cherry, peach and sour cherry J Am Soc Hort Sci 2000 125 76 80

[B46] Mnejja M Garcia-Mas J Howad W Badenes L Arús P Simple-sequence repeat (SSR) markers of Japanese plum (*prunus salicina* Lindl) are highly polymorphic and transferable to peach and almond Mol Ecol Notes 2004 4 2 163 166 10.1111/j.1471-8286.2004.00603.x

[B47] Cantini C Iezzoni AF Lamboy WF Boritzki M Struss D DNA fingerprinting of tetraploid cherry germplasm using simple sequence repeats J Am Soc Hort Sci 2001 126 2 205 209

[B48] Liu K Muse SV PowerMarker: an integrated analysis environment for genetic marker analysis Bioinformatics 2005 21 9 2128 2129 10.1093/bioinformatics/bti282 15705655

[B49] Nei M Li WH Mathematical model for studying genetic variation in terms of restriction endonucleases Proc Natl Acad Sci 1979 76 10 5269 5273 10.1073/pnas.76.10.5269 291943 PMC413122

[B50] van de Peer Y De Wachter R TREECON for windows: a software package for the construction and drawing of evolutionary trees for the microsoft windows environment Comput appl in the biosci: CABIOS 1994 10 5 569 570 7828077 10.1093/bioinformatics/10.5.569

[B51] Pritchard JK Stephens M Donnelly P Inference of population structure using multilocus genotype data Genetics 2000 155 2 945 959 10835412 10.1093/genetics/155.2.945 PMC1461096

[B52] Jakobsson M Rosenberg NA CLUMPP: a cluster matching and permutation program for dealing with label switching and multimodality in analysis of population structure Bioinformatics 2007 23 14 1801 1806 10.1093/bioinformatics/btm233 17485429

[B53] Zanetti E De Marchi M Dalvit C Cassandro M Genetic characterization of local italian breeds of chickens undergoing in situ conservation Poultry Sci 2010 89 3 420 427 10.3382/ps.2009-00324 20181856

[B54] Rosenberg NA DISTRUCT: a program for the graphical display of population structure Mol Ecol Notes 2004 4 1 137 138

[B55] Excoffier L Lischer HE Rlequin suite ver 3.5: a new series of programs to perform population genetics analyses under linux and windows Mol Ecol Resour 2010 10 3 564 567 10.1111/j.1755-0998.2010.02847.x 21565059

[B56] Peakall R Smouse PE GenAlEx 6.5: genetic analysis in excel. Population genetic software for teaching and research—an update Bioinformatics 2012 28 19 2537 2539 10.1093/bioinformatics/bts460 22820204 PMC3463245

[B57] Kruglyak L Prospects for whole-genome linkage disequilibrium mapping of common disease genes Nat Genet 1999 22 2 139 144 10.1038/9642 10369254

