# Supplementary material for: Peach genetic resources: diversity, population structure and linkage disequilibrium
Source: BMC Genet. 2013 Sep 16;14:84. doi: 10.1186/1471-2156-14-84 (PMC3848491; doi:10.1186/1471-2156-14-84)
Supplement: Additional file 1: Figure S1 — Neighbour-joining tree with bootstrap support values (>80%, based on 1,000 bootstraps) for the 658 Prunus accessions. The tree was rooted using one wild relative species Guang He Tao (Prunus mira. Koehne.) as outgroup. The colored parentheses indicate the clusters which were inferred by STRUCTURE analysis of 5 subpopulations, the subpopulation IDs are noted on the right. Accessions with different colors indicate they were assigned to corresponding subpopulations. Unstructured accessions are in red. [file 1471-2156-14-84-S1.ppt]

## Slide 1
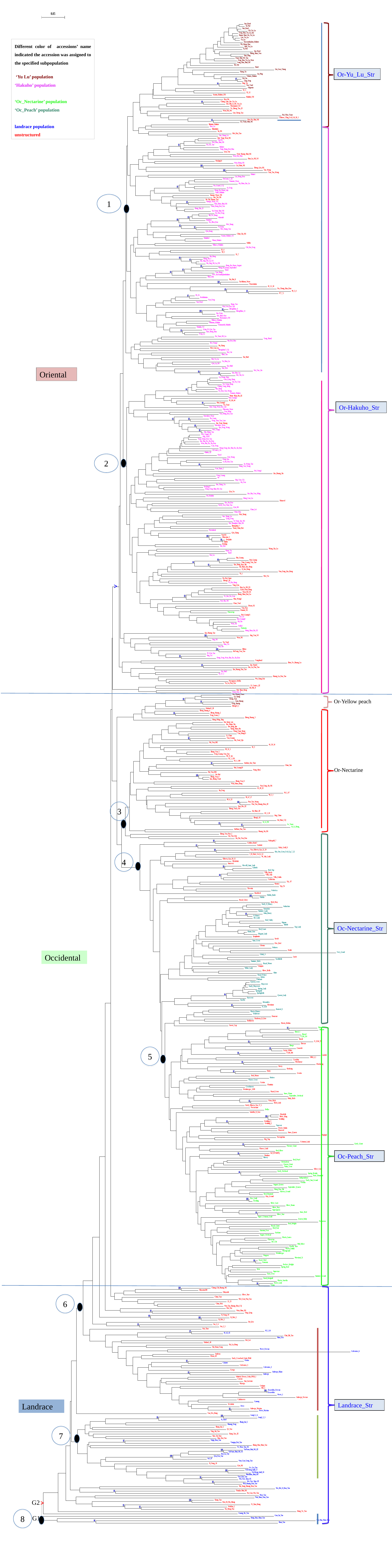

Or-Yu_Lu_Str
Or-Hakuho_Str
>
Or-Yellow peach
Or-Nectarine
Oc-Nectarine_Str
Oc-Peach_Str
Landrace_Str
>
G2
>
G1
Oriental
Occidental
Landrace
1
2
3
4
5
6
7
8
Different color of accessions’ name indicated the accession was assigned to the specified subpopulation：
 ‘Yu Lu’ population ‘Hakuho’ population
 ‘Oc_Nectarine’ population ‘Oc_Peach’ population
 landrace population unstructured
