# Supplementary material for: Peach genetic resources: diversity, population structure and linkage disequilibrium
Source: BMC Genet. 2013 Sep 16;14:84. doi: 10.1186/1471-2156-14-84 (PMC3848491; doi:10.1186/1471-2156-14-84)
Supplement: Additional file 2: Table S2 — Analysis of molecular variance (AMOVA) based on the 48 SSR loci of 587 Prunus accessions among 8 major groups by phylogenetic analysis (p < 0.05). [file 1471-2156-14-84-S2.doc]

**Supplemental table 2. Analysis of molecular variance (AMOVA) based on the 48 SSR loci of 587 *Prunus* accessions among the 8 major clusters inferred from phylogenetic tree.**

| Source of variation | d.f. | Sum of squares | Variance components | Fixation indices | Percentage of variation |
| --- | --- | --- | --- | --- | --- |
| Among population | 7 | 1779.920 | 1.79092 Va | Fst=0.19765 | 19.76 |
| Among individuals within populations | 579 | 4437.102 | 0.39302 Vb | Fis=0.05406 | 4.34 |
| Within individuals | 587 | 4037.000 | 6.87734 Vc | Fit=0.24102 | 75.90 |
| Total | 1173 | 10254.022 | 9.06129 |  |  |
