# Supplementary material for: Peach genetic resources: diversity, population structure and linkage disequilibrium
Source: BMC Genet. 2013 Sep 16;14:84. doi: 10.1186/1471-2156-14-84 (PMC3848491; doi:10.1186/1471-2156-14-84)
Supplement: Additional file 3: Table S3 — Pairwise estimates of Fst based on 48 SSRs among 8 major groups (including 587 accessions) (p < 0.05). [file 1471-2156-14-84-S3.doc]

**Supplemental table3.Pairwise estimates of Fst based on 48 SSRs among the 8 major clusters inferred from phylogenetic tree.**

|  | Cluster1 | Cluster2 | Cluster3 | Cluster4 | Cluster5 | Cluster6 | Cluster7 | Cluster8 |
| --- | --- | --- | --- | --- | --- | --- | --- | --- |
| Cluster1 | 0.0000 |  |  |  |  |  |  |  |
| Cluster2 | 0.12448 | 0.0000 |  |  |  |  |  |  |
| Cluster3 | 0.15823 | 0.21146 | 0.0000 |  |  |  |  |  |
| Cluster4 | 0.06683 | 0.16636 | 0.07373 | 0.0000 |  |  |  |  |
| Cluster5 | 0.11406 | 0.10966 | 0.29962 | 0.21954 | 0.00000 |  |  |  |
| Cluster6 | 0.15827 | 0.25373 | 0.30446 | 0.19893 | 0.26809 | 0.00000 |  |  |
| Cluster7 | 0.18109 | 0.26559 | 0.25745 | 0.19980 | 0.30935 | 0.18994 | 0.00000 |  |
| Cluster8 | 0.27747 | 0.36468 | 0.30275 | 0.28247 | 0.40110 | 0.35310 | 0.21219 | 0.0000 |

Note: Cluster1 to cluster8 represented ‘Yu_Lu’cluster, ‘Hakuho’ cluster, ‘Oriental nectarine’ cluster, ‘Occidental peach’ cluster, ‘Occidental nectarine’ cluster, ‘Occidental landrace’ cluster, ‘Oriental landrace’ cluster, ‘Wile related species’ cluster, respectively
