# Supplementary figures and images for: Peach genetic resources: diversity, population structure and linkage disequilibrium
Source: BMC Genet. 2013 Sep 16;14:84. doi: 10.1186/1471-2156-14-84 (PMC3848491; doi:10.1186/1471-2156-14-84)

## Slide 1
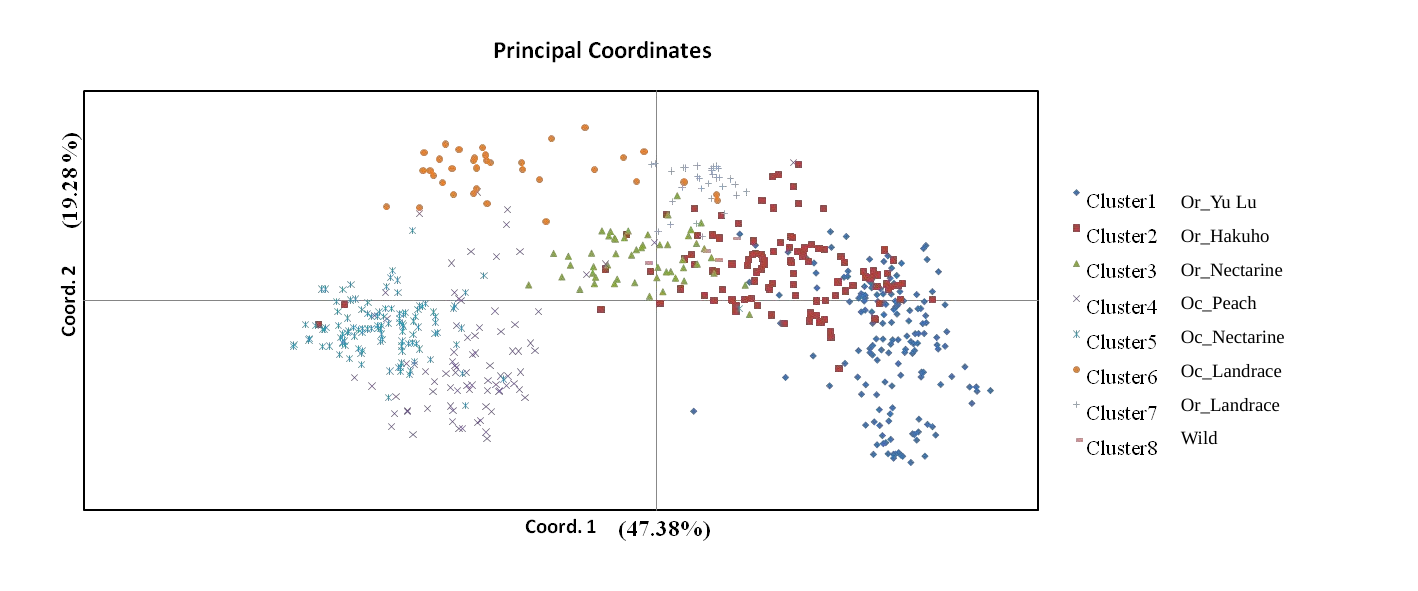

Or_Yu Lu
Or_Hakuho
Or_Nectarine
Oc_Peach
Oc_Nectarine
Oc_Landrace
Or_Landrace
Wild

Supplement: Additional file 4: Figure S2 — Principal coordinate analysis (PCoA) of 587 prunus accessions. The different colors represent the 8 major groups inferred by phylogenetic analysis. The first and second principal coordinates account for 44.28% and 21.64% of the total variation, respectively. [file 1471-2156-14-84-S4.ppt]

## Slide 1
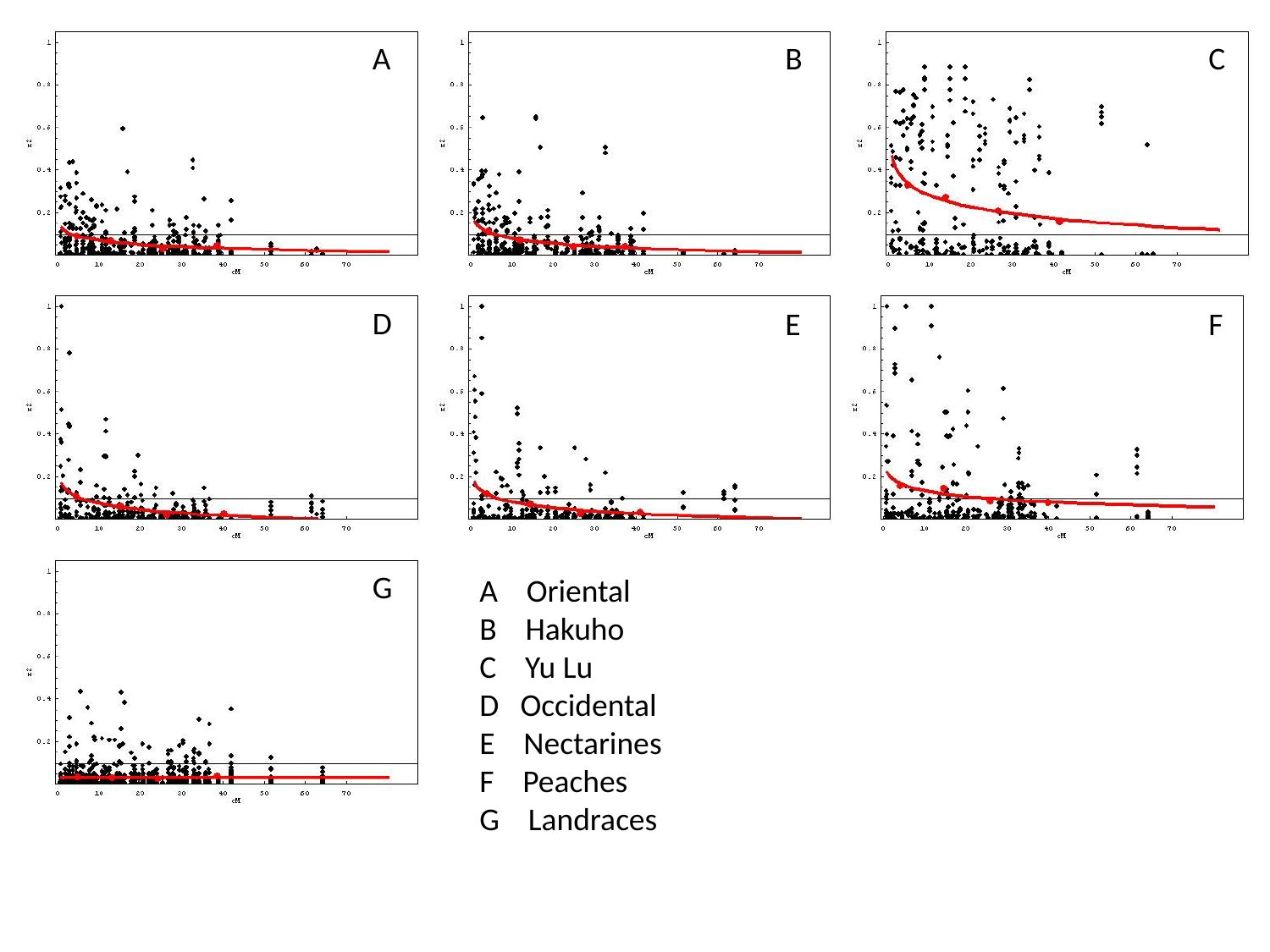

A Oriental
B Hakuho
C Yu Lu
D Occidental
E Nectarines
F Peaches
G Landraces
A
B
C
D
E
F
G

Supplement: Additional file 5: Figure S3 — LD decay plot in the subpopulations summarizing STRUCTURE and Nested STRUCTURE result. The correlation between the X and Y axis indicates the decay trend of the LD coefficient (r2) with genetic distance within intrachromosome. At the top, A, B and C show the LD level in the large ‘Oriental’ , ‘Hakuho’ and ‘Yu Lu’ subpopulations, respectively. In the middle, D, E and F show the LD level in the large ‘Occidental’ , ‘Nectarine’ and ‘Peach’ subpopulations, respectively. G shows the LD level in the ‘Landrace’ subpopulation. The horizontal line in each plot indicates r2 = 0.1. [file 1471-2156-14-84-S5.ppt]
