# Supplementary material for: Peach genetic resources: diversity, population structure and linkage disequilibrium
Source: BMC Genet. 2013 Sep 16;14:84. doi: 10.1186/1471-2156-14-84 (PMC3848491; doi:10.1186/1471-2156-14-84)
Supplement: Additional file 6: Table S1 — Detailed information on the studied cultivars including main fruit traits, pedigree, origin and geographic area in China. [file 1471-2156-14-84-S6.doc]

Supplemental Table 1 Detailed information of the studied cultivars including main fruit traits, pedigree, origin, collections and geographic area

| **Cultivar** | **Traits** | **Pedigree** | **Origin** | **Collection** | **Geographic**  **area** | **Breeding/**  **landrace** |
| --- | --- | --- | --- | --- | --- | --- |
| 01-11-8 | PWF | 96-5-4×01-33-32 | Zhengzhou | NPGR(Zhengzhou) | N | B |
| 01-39-34 | NWF | 9260×Zhong You5 | Zhengzhou | NPGR(Zhengzhou) | N | B |
| 01-42-45 | PWF | - | Zhengzhou | NPGR(Zhengzhou) | N | B |
| 01-60-44 | NWR | 26-41×Zhong You5 | Zhengzhou | NPGR(Zhengzhou) | N | B |
| 01-69-32 | NWF | Shu Guang×97-3-9 | Zhengzhou | NPGR(Zhengzhou) | N | B |
| 03-5-10 | NYF | 9260×Ai Li Mi | Zhengzhou | NPGR(Zhengzhou) | N | B |
| 05-1-139 | NYR | 99-2-54×99-43-58 | Zhengzhou | NPGR(Zhengzhou) | N | B |
| 05-1-140 | NWR | 99-2-54×99-43-58 | Zhengzhou | NPGR(Zhengzhou) | N | B |
| 05-3-16 | NWF | Seedling selection from 01-42-45 | Zhengzhou | NPGR(Zhengzhou) | N | B |
| 21-25 | PWR | - | Zhengzhou | SWU | N | B |
| 21Centry_SX | PWR | - | Hebei | SAAS | N | B |
| 22Centry_ZZ | PWR | Dan Gui × Xue Tao | Hebei | NPGR(Zhengzhou) | N | B |
| 36-3 | PWR | - | Zhengzhou | SWU | N | B |
| 63-17-1 | PWR | Yu Lu × Wasasuimitsu | Jiangsu | NPGR(Jiangsu) | SE | B |
| 76-1 | PWR | - | Chongqing | SWU | SW | B |
| 79-6-9 | PWR | - | Shanxi | NPGR(Zhengzhou) | N | B |
| 87-7-1 | PWR | Seedling selection from(Hakuho × Shou Xing) | Zhengzhou | NPGR(Zhengzhou) | N | B |
| 89-4-32 | PWR | Zhao Hui× Shuang Fu | Zhengzhou | NPGR(Zhengzhou) | N | B |
| 94 | PWR | - | Chongqing | SWU | SW | B |
| 94-1-47 | NWR | - | Zhengzhou | NPGR(Zhengzhou) | N | B |
| 96-5-1 | PWR | 87-7-1×Ai Li Mi | Zhengzhou | NPGR(Zhengzhou) | N | B |
| 96-5-4 | PWR | 87-7-1×Ai Li Mi | Zhengzhou | NPGR(Zhengzhou) | N | B |
| 99-31-5 | NWR | Zhong You5×SD9238 | Zhengzhou | NPGR(Zhengzhou) | N | B |
| 99-37-46 | NWR | 89-1-28×Zhong you5 | Zhengzhou | NPGR(Zhengzhou) | N | B |
| 99-47-17 | NWR | 94-1-47×Zhong You4 | Zhengzhou | NPGR(Zhengzhou) | N | B |
| A Bu Bai Tao | PWR | - | Japan | SWU | J | L |
| Ai Feng | PWR | - | unknown | SWU | SW | L |
| Ai Li Hong | NYR | Zao Hong2×Sumfr | Zhengzhou | NPGR(Zhengzhou) | N | B |
| Ai Li Mi | NYR | - | Zhengzhou | NPGR(Zhengzhou) | N | B |
| Akatsuki | PWR | Hakuho Mutation × Baitao | Japan | FHPRI | J | B |
| An Nong Shui Mi | PWR | mutant of SunagoWase | Anhui | NPGR(Zhengzhou) | SE | B |
| An Ping | PWR | - | unknown | FHPRI | SE | L |
| Asama Hakuto_FH | PWR | Gaoyangbaitao Mutation | Japan | FHPRI | J | B |
| Asama Hakuto_NJ | PWR | Gaoyangbaitao Mutation | Japan | NPGR(Jiangsu) | J | B |
| Bai Gen Bai Tao | PWR | Unknown | Japan | FHPRI | J | L |
| BaiHua Shan Tao *(Prunus davidiana)* | PWR | - | unknown | NPGR(Jiangsu) | N | W |
| Bai Hua Shui Mi | PWR | Seedling Selection of Chinese Cling | unknown | NPGR(Jiangsu) | SE | L |
| Bai Lu | PWR | Unknown | Japan | FHPRI | J | L |
| Bai Lu Tao | PWR | - | Shanxi | SAAS | N | L |
| Bai Mao Yuan | PWR | - | Jiangsu | NPGR(Jiangsu) | SE | L |
| Bai Sha Mi | PWR | - | Shanxi | SAAS | N | L |
| Bai Tao | PWR | Seedling Selection of Chinese Cling | Japan | NPGR(Jiangsu) | J | B |
| BaiTian | NWR | - | Shanxi | SAAS | N | L |
| Bao Lu | PWR | - | unknown | SWU | - | L |
| Bei Jing | PWR | - | Beijing | FHPRI | N | L |
| Bei Jing27 | PWR | Unknown | Beijing | SWU | N | B |
| Bei Lei | PWR | - | Canada | NPGR(Zhengzhou) | O | B |
| Bei Nong1 | PWR | Okubo ×Amsden June | Beijing | NPGR(Jiangsu) | N | B |
| Bei Nong2 | PWR | Seedling Selection of Okayamawase | Beijing | NPGR(Jiangsu) | N | B |
| Beijing14 | PWR | - | unknown | NPGR(Zhengzhou) | N | B |
| Ben Di Pin Zhong | PWR | - | Chongqing | SWU | SW | L |
| Chang Chi Huang Mi | PWR | - | Jiangsu | NPGR(Jiangsu) | SE | L |
| Chang Ling Zao Yu Lu | PWR | Local Cultivar | Zhejiang | NPGR(Zhengzhou) | SE | L |
| Chao Yue1 | NWR | - | Shanxi | SAAS | N | L |
| Cheng Yan | PYR | Seedling selection from Zao Sheng Huang Jin | Dalian | NPGR(Zhengzhou) | NE | B |
| Chi BaiFeng | PWR | Hakuho Mutation | Zhejiang | FHPRI | SE | B |
| Chi Yu Lu | PWR | Yu Lu Mutation | Zhejiang | FHPRI | SE | B |
| Chong Yang Hong | PWR | Mutant of Okubo | Hebei | SAAS | N | B |
| Chui Zhi Tao | PWR | Unknown | Zhengzhou | NPGR(Zhengzhou) | N | L |
| Chun Hua | PWR | BeiNong 2 × Chun Lei | Shanghai | NPGR(Jiangsu) | SE | B |
| Chun Lei | PWR | Sunagowase × Bai Xiang Lu | Shanghai | NPGR(Jiangsu) | SE | B |
| Chun Mei | PWR | (Zao hong2×Flatpeach)×(Rui guang3×May fire) | Zhengzhou | NPGR(Zhengzhou) | N | B |
| Chun Xia Mi | PWR | Shen Zhou Mi Tao × Yu Hua Lu | Beijing | NPGR(Jiangsu) | N | B |
| Chun Xue | PWR | - | USA | SWU | O | B |
| Chun Yan | PWR | ZaoFeng Wang × Kurakatowase | Shandong | NPGR(Jiangsu) | N | B |
| Da Bai Feng | PWF | Selected from Hakuho | Zhejiang | FHPRI | SE | B |
| Da Bai Suan | NWR | - | unknown | NPGR(Zhengzhou) |  | L |
| Da Chong Hua Ban | PWR | - | Chongqing | SWU | SW | L |
| Da Guan | PWR | Selected from Nunomewase | Zhejiang | ZJU | SE | B |
| Da Guan1_CQ | PWR | Selected from Nunomewase | Zhejiang | SWU | SE | B |
| Da Guo Wu Tao | PWR | - | Shandong | SWU | N | L |
| Da Hong Hua | PWR | Local Cultivar | Jiangsu | NPGR(Jiangsu) | SE | L |
| Da Hong Pao | PRR | - | Hubei | NPGR(Zhengzhou) | SE | L |
| Da Jie Tao | PWR | - | unknown | NPGR(Jiangsu) | - | L |
| Da Tuan Mi Lu | PWR | Seedling Selection of Tai Chang Shui Mi | Shanghai | NPGR(Jiangsu) | SE | B |
| Da Zhen Bao | PWR | - | Japan | NPGR(Zhengzhou) | J | L |
| Da Guan 1_ZZ | PWR | Selected from Nunomewase | Zhejiang | NPGR(Zhengzhou) | SE | B |
| Dalian12-28 | PYR | - | Dalian | NPGR(Zhengzhou) | NE | B |
| Dalian1-49 | PYR | - | Dalian | NPGR(Zhengzhou) | NE | B |
| Dan Mo_SX | NYR | 81-3-76（Jing Yu×Njn76）×Zao Hong2 | Beijing | SAAS | N | B |
| Dan Mo_ZZ | NYR | 81-3-76（Jing Yu×Njn76）×Zao Hong3 | Beijing | NPGR(Zhengzhou) | N | B |
| Denjiulo | PWR | - | Japan | NPGR(Jiangsu) | J | L |
| Dixon_SX | PWR | - | USA | SAAS | O | B |
| Dixon_ZZ | NYR | - | USA | NPGR(Zhengzhou) | O | B |
| Dong Fang Hong | PWR | HuaGuang Mutation | Shan'xi | SWU | SW | B |
| Dong FengShui Mi Tao | PWR | - | Shanxi | SAAS | N | L |
| Dong Feng Wan Shu Da JiuBao | PWR | Okubo Mutation | Shanxi | SAAS | N | B |
| Dong FengZaoShu Da JiuBao | PWR | Okubo Mutation | Shanxi | SAAS | N | B |
| Du Hakuho | PWR | - | Japan | NPGR(Jiangsu) | J | B |
| Duan Yu | PWR | Hatsukami ×Amsden | Shanxi | SAAS | N | B |
| E Tao1 | PWR | Local Cultivar | Hubei | NPGR(Jiangsu) | S | L |
| Early-Fen Tian Tao | PWR | Local Cultivar | Shanxi | SAAS | N | L |
| Fantasia | NYR | Cold King × Red King | USA | SAAS | O | B |
| FeiChengbaili 10 | PWR | Local Cultivar | Shandong | NPGR(Zhengzhou) | N | L |
| FeiChengbaili 17 | PWR | Local Cultivar | Shandong | NPGR(Zhengzhou) | N | L |
| FengBai | PWR | Seedling Selection of Okubo | Dalian | NPGR(Jiangsu) | NE | B |
| Feng Guan 2 | PYR | Guan Tao5 × LianHuang | Zhejiang | NPGR(Zhengzhou) | SE | B |
| FengGuang | NWR | - | Shanxi | SAAS | N | B |
| FengGuang You Tao | NWR | Unknown | Japan | SAAS | J | B |
| FengHua Yu Lu Wan | PWR | Mutant of Yu Lu | Zhejiang | NPGR(Jiangsu) | SE | B |
| FengHua Yu Lu Zao | PWR | Mutant of Yu Lu | Zhejiang | NPGR(Jiangsu) | SE | B |
| Feng Huang | PYR | Seeding Selection of Zao Sheng Huang Jin | Dalian | NPGR(Zhengzhou) | NE | B |
| Feng Huang Shui Mi | PWR | Bud Mutant of Wuxi Cling | Jiangsu | NPGR(Jiangsu) | SE | B |
| Feng Lu | PWR | Seedling Selection of Hakuho | Shanghai | NPGR(Jiangsu) | SE | B |
| FengTian Tao-Late | PWR | Local Cultivar | Shanxi | SAAS | N | L |
| Flavor Top | NYR | Seedling Selection from Fairtime | USA | SAAS | O | B |
| Fu Dao Tao Wang | NWR | - | Shanxi | SAAS | N | L |
| FuanShui Mi Tao | PWR | - | Fujian |  | S | L |
| Gan Su Tao *(PrunusKansuensis)* | PWR | - | Gansu | NPGR(Jiangsu) | NW | W |
| GanXuan 2 | PYR | - | Gansu | NPGR(Zhengzhou) | NW | L |
| GaoFeng | PWR | - | Shanxi | SAAS | N | L |
| GeGu Tao | PWR | - | Hubei | NPGR(Zhengzhou) | SE | L |
| Golden Baby7 | PYR | (Lemon Free×P.l.35201)×NJ196 | USA | NPGR(Zhengzhou) | O | B |
| Guang He Tao *(Prunus Mira)* | PWR | - | Tibet | NPGR(Jiangsu) | NW | W |
| Guo Qing Hong | NWR | - | Shanxi | SAAS | N | L |
| Ha Lu Hong | PRR | - | USA | NPGR(Zhengzhou) | O | B |
| Hakuho_FH | PWR | Hakuto × Tachibanawase | Japan | NPGR(Jiangsu) | J | B |
| Hakuho_NJ | PWR | Hakuto × Tachibanawase | Japan | NPGR(Jiangsu) | J | B |
| Hakuho1 | PWR | Hakuho Mutation | Japan | FHPRI | J | B |
| Hakuho2 | PWR | Hakuho Mutation | Japan | FHPRI | J | B |
| Hakuri | PWR | Okubo × Fei Cheng Tao | Japan | FHPRI | J | B |
| Hakuto | PWR | Seedling Selection of Chinese Cling | Japan | NPGR(Jiangsu) | J | B |
| Han Lu Mi_NJ | PWR | Seedling Selection of Chinese Cling | Shandong | NPGR(Jiangsu) | N | B |
| Han Lu Mi_SX | PWR | Seedling Selection of Chinese Cling | Shandong | SAAS | N | B |
| Hang Mi 1 | PWR | SunagoWase ×Yu Hua Lu | Zhejiang | NPGR(Zhengzhou) | SE | B |
| Hatsukami | PWR | Introduced from Japan | Japan | NPGR(Jiangsu) | J | L |
| HeYang You Tao | NYR | - | Shan'xi | NPGR(Zhengzhou) | NW | L |
| HikawaHakuho_FH | PWR | Unknown | Japan | FHPRI | J | B |
| HikawaHakuho_NJ | PWR | Unknown | Japan | NPGR(Jiangsu) | J | B |
| Hong Bao Shi | NYR | - | unknown | NPGR(Zhengzhou) |  | L |
| Hong Bu Ruan-August | PWR | Local Cultivar | Shanxi | SAAS | N | L |
| Hong Bu Ruan-July | PWR | Local Cultivar | Shanxi | SAAS | N | L |
| Hong Bu Ruan-September | PWR | Local Cultivar | Shanxi | SAAS | N | L |
| Hong Gan Lu | PWR | Seedling Selection of Hong Tian Tao | Dalian | NPGR(Jiangsu) | NE | B |
| Hong Hua Shan Tao *(Prunus davidiana)* | PWR | - | unknown | NPGR(Jiangsu) | N | W |
| Hong Ming Xing | PYR | Feng Huang × Guang Tao14 | Shan'xi | SWU | NW | B |
| Hong ShaZi | PWR | - | unknown | NPGR(Zhengzhou) |  | L |
| Hong Shan Hu_SX | NWR | Qiu Yu×NJN76 | Beijing | SAAS | N | B |
| Hong Shan Hu_ZZ | NWR | Qiu Yu×NJN77 | Beijing | NPGR(Zhengzhou) | N | B |
| Hong Tao Wang | PWR | - | Liaoning | FHPRI | NE | L |
| Hong Ye Tao | - | - | unknown | SWU | N | L |
| Hong Yue | PWR | Bai Tao × Hakuho | Japan | SWU | J | B |
| Hu Jing Mi Lu_FH | PWR | Bud Mutant of Hahuho | Jiangsu | NPGR(Jiangsu) | SE | B |
| Hu Jing Mi Lu_NJ | PWR | Bud Mutant of Hahuho | Jiangsu | NPGR(Jiangsu) | SE | B |
| Hu You 002_ZZ | NWR | Ruiguang3×Mayfire | Shanghai | NPGR(Zhengzhou) | SE | B |
| Hu You 004 | NYR | ‘25-17’×Mayfire | Shanghai | NPGR(Zhengzhou) | SE | B |
| Hu You 2_ZJU | NWR | Ruiguang3×Mayfire | Shanghai | ZJU | SE | B |
| Hua Wang2 | NWR | - | Shanxi | SAAS | N | L |
| Hua Yu | PWR | Jiang Yu × RuiGuang 7 | Beijing | NPGR(Jiangsu) | N | B |
| Hua Yu Lu | PWR | Mutant of Yu Lu | Zhejiang | NPGR(Jiangsu) | SE | B |
| Huang Jin Mi | PYR | - | Shandong | NPGR(Zhengzhou) | N | L |
| Huang Lu Pan Tao | PYF | Local Cultivar | Zhejiang | FHPRI | SE | L |
| Hui Yu Lu | PWR | Zhaohui×Yuhualu | Jiangsu | NPGR(Jiangsu) | SE | B |
| Italia1 | NYR | - | Italy | SAAS | O | B |
| Ji Zao Hong | PWR | - | Zhejiang | ZJU | SE | L |
| Jia Na Yan | PWR | Okubo×Okitsu | Japan | SAAS | J | B |
| Jian Ding | PWR | - | Zhejiang | FHPRI | SE | B |
| Jian Yang Wan Bai Tao | PWR | Seedling Selection from Shang Hai Shui Mi | Sichuan | SWU | SW | B |
| JiazhouZaoTian | PWR | - | USA | NPGR(Zhengzhou) | O | B |
| Jin Feng | PYR | - | Zhengzhou | NPGR(Zhengzhou) | N | B |
| Jin Hui | NYR | Rui Guang2×Armking | Zhengzhou | NPGR(Zhengzhou) | N | B |
| Jin Jun | PWR | Unknown | Zhejiang | FHPRI | SE | L |
| Jin Qiu | PYR | Yang Quan Rou Tao×Star | Shanxi | SAAS | N | B |
| Jin Shan Zao Hong | NWR | Mutant of Zao Hong Bao Shi | Jiangsu | FHPRI | SE | B |
| Jin Shan Zao Lu | PWR | Seedling Selection of Hakuho | Jiangsu | NPGR(Jiangsu) | SE | B |
| Jin Shi Ji | PWR | Dan Gui×Xue Tao | Hebei | SWU | N | B |
| Jin Shuo_CQ | NYR | - | unknown | SWU | N | B |
| Jin Shuo_ZZ | NYR | Zao Hong2×ShuGuang | unknown | NPGR(Zhengzhou) | N | B |
| Jin Xia | NWR | Okubo×Okitsu | Shanxi | SAAS | N | B |
| Jin Xia You Pan | NYF | Xia Guang×NF | Jiangsu | NPGR(Zhengzhou) | SE | B |
| Jin Xiang | PYR | Bei Nong2×60-24-7 | Shanghai | NPGR(Zhengzhou) | SE | B |
| Jin Xiu Huang Tao | PYR | BaiHua × Yunshu1 | Shanghai | ZJU | SE | B |
| Jin You | PWR | Jing Yan×Xia Zhi Tao | Chongqing | SWU | SW | B |
| Jing Chun | PWR | Seedling Selection from Zao Sheng Huang Jin | Beijing | SWU | N | B |
| Jing Mi | PWR | LvHua 1 × Okitsu | Beijing | NPGR(Jiangsu) | N | B |
| Jing Tang Wan Mi | PWR | Seedling Selection of Denjyoro | Hebei | NPGR(Jiangsu) | N | B |
| Jing Yan_CQ | PWR | Okubo×Lv Hua5 | Beijing | SWU | N | B |
| Jing Yan-NJ | PWR | LvHua 5 × Okubo | Beijing | NPGR(Jiangsu) | N | B |
| Jing Yu | PWR | Okubo×Okitsu | Beijing | NPGR(Zhengzhou) | N | B |
| Jing Yu14 | PWR | Okubo×Okitsu | Beijing | IFPBAAFS | N | B |
| JinShan Pan Tao | PWF | - | Shanghai | NPGR(Zhengzhou) | SE | L |
| Ju Wang Tao | PWR | - | Anhui | FHPRI | SE | L |
| KawanakajimaHakuto | PWR | - | Japan | FHPRI | J | B |
| KurakatoWase_FH | PWR | Seedling from（Tuscan×Hong Tao） | Japan | FHPRI | J | B |
| KurakatoWase_NJ | PWR | Seedling from（Tuscan×Hong Tao） | Japan | NPGR(Jiangsu) | J | B |
| Lai Hong Rou | PWR | - | Shanxi | SAAS | N | L |
| Lai Shan Mi | PWR | Seedling Selection | Shandong | NPGR(Jiangsu) | N | B |
| Lao Yu Lu | PWR | Local Cultivar | Zhejiang | FHPRI | SE | L |
| Le Yuan | NYR | - | Zhengzhou | NPGR(Zhengzhou) | N | L |
| Li Chun | NWR | Rui guang3×Mayfire | Beijing | SWU | N | B |
| Lian Huang | PYR | Seedling selection from Zao Sheng Huang Jin | Dalian | NPGR(Zhengzhou) | NE | B |
| Lin Cheng Tao | PWR | Local Cultivar | Shandong | NPGR(Zhengzhou) | N | L |
| Lin Feng | PWR | - | Japan | FHPRI | J | L |
| Lin Guo Chang | PWR | Unknown | Zhejiang | FHPRI | SE | L |
| Lin Jia | PWR | Unkown | Japan | FHPRI | J | L |
| Liu YueBai | PWR | Local Cultivar | Hubei | NPGR(Zhengzhou) | N | L |
| Long HuaShui Mi | PWR | Unknown | Shanghai | NPGR(Jiangsu) | SE | L |
| Long1-2-3 | PYR | Local Cultivar | Gansu | NPGR(Zhengzhou) | NW | L |
| Long1-2-4 | PYR | Local Cultivar | Gansu | NPGR(Zhengzhou) | NW | L |
| Lu Wang Xian | PWR | - | Japan | SAAS | J | L |
| Lu Xiang | PYR | Seedling selection from Zao Sheng Huang Jin | Dalian | NPGR(Zhengzhou) | NE | B |
| Mai Huang Pan Tao | PYF | - | Zhengzhou | NPGR(Zhengzhou) | N | L |
| Mai Xiang | PWR | Okubo×Amsden June | Beijing | NPGR(Jiangsu) | N | B |
| Mang Xia Lu | PWR | BaiHua× Hatsukami | Jiangsu | SAAS | SE | B |
| Mao Tao *(Prunuspersica)* | PWR | Wild Tape Used For Root Stock | unknown | NPGR(Jiangsu) | - | WL |
| Matsumori_FH | PWR | Seedling Selection of Hakuho | Japan | FHPRI | J | B |
| Matsumori_NJ | PWR | Seedling Selection of Hakuho | Japan | NPGR(Jiangsu) | J | B |
| Mei Bai Tao | PWR | Shimizu Seedling | Korea | FHPRI | K | B |
| Mei Gui Lu | PWR | SunagoWase×Yu Hua Lu | Zhejiang | NPGR(Zhengzhou) | SE | B |
| Mei Guo Pan Tao | PWF | - | USA | FHPRI | O | B |
| Mei Xiang | PWR | Yuzora Mutation | Japan | SWU | J | B |
| Mei Xiang Tao | PWR | - | unknown | FHPRI | - | B |
| Mei Yu | NWR | - | JingYu'×'RuiGuang7' | Beijing | N | L |
| Nan Can Gong Tao | PWR | Mutant | Chongqing | SWU | SW | B |
| Nan Fang Jin MI | NYR | - | unknown | NPGR(Zhengzhou) |  | L |
| Nan Fang Zao Hong | PWR | - | Zhengzhou | SWU | N | L |
| Nan Shan Tian Tao | PWR | Local Cultivar | Guangdong | Shenzhen | S | L |
| Nan Tao Wang | PWR | - | Shanxi | SAAS | N | L |
| New KawanakajimaHakuto | PWR | KawanakajimaHakuto Mutation | Zhejiang | FHPRI | SE | B |
| NkazuHakuto | PWR | Hakuto Seedling | Japan | FHPRI | J | B |
| NongShen | PWR | - | France | NPGR(Zhengzhou) | O | N |
| Nunomewase | PWR | Unknown | Japan | NPGR(Jiangsu) | J | B |
| Ohayashi | PWR | Unknown | Japan | NPGR(Jiangsu) | J | L |
| OhdamaHakuho | PWR | Tachibana×Hakuto | Japan | FHPRI | J | B |
| Okayama 3 | PWR | Doyou×Susquehanna | Japan | NPGR(Jiangsu) | J | B |
| Okayama 500 | PWR | King×Red Birdcling | Japan | NPGR(Jiangsu) | J | B |
| Okayamawase | PWR | Seedling Selection of Shang Hai Cling | Japan | NPGR(Jiangsu) | J | B |
| Okitsu | NYR | Precoce de Croncels× Lord Napier | Japan | NPGR(Zhengzhou) | J | B |
| Okubo_NJ | PWR | Seedling Selection of Hakuto | Japan | NPGR(Jiangsu) | J | B |
| Okubo_ZZ | PWR | - | Japan | NPGR(Zhengzhou) | J | B |
| Ougento | PYR | - | Japan | Jiaxing | J | B |
| Pan Tao Huang Hou_CQ | PWF | Zaohong2 × ZaoLuPanTao | Zhengzhou | SWU | N | L |
| Pan Tao Huang Hou_ZZ | PWF | Zaohong2 × ZaoLuPanTao | Zhengzhou | NPGR(Zhengzhou) | N | L |
| Pan Tao Wang | PWF | - | unknown | NPGR(Zhengzhou) | N | L |
| Pi Qiu Tao | PWR | Local Cultivar | Chengdu | SWU | SW | L |
| Qi Tao | PWR | Local Cultivar | Gansu | NPGR(Zhengzhou) | NE | L |
| Qi Yuan Shui Mi | PWR | Local Cultivar | Zhejiang | NPGR(Jiangsu) | SE | L |
| Qian Dai Ji | PWR | Hakuto×Hakuho | Japan | FHPRI | J | B |
| QianNian Hong | NYR | Hakuho×May fire | Zhengzhou | SAAS | N | B |
| Qin Lu Mi | PWR | - | unknown | SWU | - | L |
| Qin Mi | PWR | Okubo×Chun Lei | Shan'xi | SWU | NW | B |
| Qin Wang | PWR | Seedling Selection from Okubo | Shan'xi | SWU | NW | B |
| Qing Feng | PWR | - | Beijing | NPGR(Zhengzhou) | N | B |
| Qing Pi Qiu Tao | PWR | - | Zhejiang | NPGR(Jiangsu) | SE | L |
| Qiu Fen Tao | PWR | Local Cultivar | Shanxi | SAAS | N | L |
| Qiu Kong | PWR | Unknown | Japan | FHPRI | J | B |
| Qiu Mi | PWR | Local Cultivar | Shan'xi | NPGR(Zhengzhou) | NW | L |
| Qiu Xiang | PWR | - | Beijing | NPGR(Zhengzhou) | N | B |
| Qiu Yu | NWR | Okubo×Okitsu | Beijing | SAAS | N | B |
| QiuYue | PWR | - | unknown | NPGR(Zhengzhou) |  | L |
| Red Shimizu | PWR | - | Japan | SWU | J | L |
| Ren He | PWR | - | Japan | FHPRI | J | B |
| RenPuShui Mi | PWR | - | Zhejiang | NPGR(Jiangsu) | SE | L |
| Ri Ai | PWR | Unknown | Japan | FHPRI | J | L |
| RuiGuang | NWR | - | Beijing | NPGR(Zhengzhou) | N | B |
| Rui Guang11 | NWR | Jing Yu×Njn76 | Beijing | SAAS | N | B |
| Rui Guang19 | NWR | Legrand×（Jingyu×Njn76） | Beijing | SWU | N | B |
| Rui Guang2 | NYR | Jing Yu×Njn76 | Beijing | IFPBAAFS | N | B |
| Rui Guang3 | NWR | Jing Yu×NJN76 | Beijing | NPGR(Zhengzhou) | N | B |
| Rui Guang8 | NWR | - | Beijing | SAAS | N | B |
| Rui Shan Hong | PWR | - | unknown | SWU | SW | L |
| Run Zhou Shui Mi | PWR | Unknown | Jiangsu | NPGR(Jiangsu) | SE | L |
| San Bao1 | PWR |  | Zhejiang | FHPRI | SE | B |
| San Bao2 | PWR | - | Zhejiang | FHPRI | SE | B |
| San Bao3 | PWR | - | Zhejiang | FHPRI | SE | B |
| San Bao4 | PWR | Unknown | Zhejiang | FHPRI | SE | B |
| Sekiho | PWR | - | Japan | NPGR(Jiangsu) | J | L |
| Sha Hong | NWR | - | Shanxi | SAAS | N | L |
| Sha Hong Tao | PWR | Bud mutatant from KurakatoWase | Shanxi | NPGR(Zhengzhou) | NW | B |
| Shan Tao *(PrunusDavidiana)* | PWR | - | unknown | NPGR(Jiangsu) | N | W |
| Shang Shan Da Yu Lu | PWR | Seedling Selection of Yu Lu | Zhejiang | FHPRI | SE | B |
| Shang Shan You | PWR | Unknown | Zhejiang | FHPRI | SE | B |
| ShenZhou Mi Tao | PWR | Local Cultivar | Hebei | NPGR(Zhengzhou) | N | L |
| Shimizu Hakuto | PWR | Unknown | Japan | FHPRI | J | B |
| Shinokubo | PWR | - | Japan | NPGR(Jiangsu) | J | B |
| Shinvhong | PWR | - | Shan'xi | SWU | N | L |
| Shou Fen | PWR | - | unknown | NPGR(Zhengzhou) |  | L |
| Shu Guang | NWR | Legrant×Rui Guang2 | Zhengzhou | NPGR(Zhengzhou) | N | B |
| Shuang Feng | PWR | Zao Xiang Yu×Okubo | Beijing | NPGR(Zhengzhou) | N | B |
| Silver Star | PWR | - | Japan | NPGR(Jiangsu) | J | L |
| Sunago Wase_FH | PWR | Seedling Selection of Okubo | Japan | NPGR(Jiangsu) | J | B |
| Sunago Wase_NJ | PWR | Seedling Selection of Okubo | Japan | SAAS | J | B |
| Sx1-07 | PWR | - | Shanxi | SAAS | N | L |
| Sxz-2-4 | PRR | - | Shanxi | SAAS | N | L |
| Sxz-2-5 | PRR | - | Shanxi | SAAS | N | B |
| Tachibana Wase | PWR | Seedling Selection of Denjyoro | Japan | NPGR(Jiangsu) | J | B |
| Tai Gu Rou Tao | PWR | Local Cultivar | Shanxi | SAAS | N | L |
| Tai Yang Hong | NWR | - | Shanxi | SAAS | N | L |
| TaiYuan Shui Mi_SX | PWR | Local Cultivar | Shanxi | NPGR(Zhengzhou) | N | L |
| TaiYuan Shui Mi_ZZ | PWR | Local Cultivar | Shanxi | NPGR(Zhengzhou) | N | L |
| Takei Hakuho | PWR | Hakuho Mutation | Japan | FHPRI | J | B |
| Tang Hang Bai Hua | PWR | Seedling Selection of Chinese Cling | Shanghai | NPGR(Jiangsu) | SE | B |
| Tao Wang | PWR | - | unknown | NPGR(Zhengzhou) |  | L |
| Tao Yan1 | PWR | Unknown | Zhejiang | FHPRI | SE | B |
| Te Zao Da Guo | NWR | - | Shanxi | SAAS | N | L |
| Tianjin Shui Mi | PWR | Local Cultivar | Tianjin | NPGR(Zhengzhou) | N | L |
| Wan Bai Hua | PWR | Seedling Selection of Chinese Cling | Sichuan | NPGR(Jiangsu) | SW | B |
| Wan Bai Mi | PWR | Wu Yun×Hakuho | Jiangsu | NPGR(Zhengzhou) | SE | B |
| Wan Hong Mi | PWR | Wan Xiang × Okubo | Hebei | NPGR(Jiangsu) | N | B |
| Wan Jin | NYR | Manuela Deluca Mutation | Shanxi | SAAS | N | B |
| Wan Mi_NJ | PWR | Unknown | Beijing | NPGR(Jiangsu) | N | B |
| Wan Mi_SX | PWR | Unknown | Beijing | NPGR(Jiangsu) | N | B |
| Wan Shou Hong | NYR | 81-4-10×Fantacia | Shandong | SWU | N | B |
| Wan Shu Da Jiu Bao | PWR | Okubo Mutation | Shanxi | SAAS | N | B |
| Wan Shuo Mi | PWR | Wan Shu Shui Mi × Fei Cheng Tao | Jiangsu | NPGR(Jiangsu) | SE | B |
| Wasesimizu | PWR | Seeding Selection of Chinese Cling | Japan | NPGR(Jiangsu) | J | B |
| Wei Yang 2_ZZ | PWR | - | Shan'xi | NPGR(Zhengzhou) | NW | L |
| Wei Yang2_SX | PWR | - | Shan'xi | SAAS | NW | L |
| Wu Hei Ji Rou Tao | PRR | - | Shandong | NPGR(Zhengzhou) | N | L |
| Wu Yue Jin | PWR | Unknown | Zhengzhou | SWU | N | L |
| Wu Yue Xian_SX | PWR | Local Cultivar | Beijing | SAAS | N | L |
| Wu Yue Xian_ZZ | PWR | Local Cultivar | Beijing | NPGR(Zhengzhou) | N | L |
| WuHan 2 | PRR | - | Shan'xi | NPGR(Zhengzhou) | NW | L |
| WuJiang Bai | PWR | Local Cultivar | Jiangsu | NPGR(Jiangsu) | SE | L |
| X1-4 | PWR | Yu Lu ×Hu Jing Mi Lu | Zhejiang | FHPRI | SE | B |
| X1-7 | PWR | Yu Lu ×Hu Jing Mi Lu | Zhejiang | FHPRI | SE | B |
| X2-12 | PWR | Shang Shan Da Yu Lu×Hu Jing Mi Lu | Zhejiang | FHPRI | SE | B |
| X2-5 | PWR | Shang Shan Da Yu Lu×Hu Jing Mi Lu | Zhejiang | FHPRI | SE | B |
| X4-1 | PWR | Yu Lu ×Hu Jing Mi Lu | Zhejiang | FHPRI | SE | B |
| Xi Jiao3 | PWR | Seedling from Shanxi | Shan'xi | NPGR(Zhengzhou) | NW | L |
| Xi Mei 1 | PWR | - | Shan'xi | NPGR(Zhengzhou) | NW | L |
| Xi Mei 2 | PWR | - | Shan'xi | NPGR(Zhengzhou) | NW | L |
| Xi Nong 18 | PWR | Xiong Nong Shui Mi×Mei Xian Dong Tao | Shan'xi | NPGR(Zhengzhou) | NW | B |
| Xi Nong 19 | PWR | Xiong Nong Shui Mi×Mei Xian Dong Tao | Shan'xi | NPGR(Zhengzhou) | NW | B |
| Xi Nong Zao Mi | PWR | Xiong Nong Shui Mi×Xin Duan Yang | Shan'xi | NPGR(Zhengzhou) | NW | B |
| Xi Pu1 | PWR | Unknown | Zhejiang | FHPRI | SE | B |
| Xi Pu4 | PWR | Unknown | Zhejiang | FHPRI | SE | B |
| Xi_An1 | PWR | - | Shan'xi | FHPRI | NW | L |
| Xi_An2 | PWR | - | Shan'xi | FHPRI | NW | L |
| Xi_An3 | PWR | - | Shan'xi | FHPRI | NW | L |
| Xia Cui | PWR | Yu Hua2×[(Bai hua×Tachibana Wase)×Zhao xia] | Jiangsu | NPGR(Zhengzhou) | SE | B |
| Xia Hui 8 | PWR | - | Jiangsu | NPGR(Zhengzhou) | SE | B |
| Xia Hui1 | PWR | Zhao Hui × Zhao Xia | Jiangsu | NPGR(Jiangsu) | SE | B |
| Xia Hui5 | PWR | Zhao Hui ×(Yu Lu × Zao Sheng Shui Mi | Jiangsu | NPGR(Jiangsu) | SE | B |
| Xia Hui6 | PWR | Zhao Hui × Yu Hua Lu | Jiangsu | NPGR(Jiangsu) | SE | B |
| Xiang Shan Hu | NWR | Qiu Yu×Njn76 | Beijing | SWU | N | B |
| Xiang Tao_CQ | PWR | Local Cultivar | Dalian | SWU | NE | L |
| Xiang tao_ZZ | PWR | Local Cultivar | Dalian | NPGR(Zhengzhou) | NE | L |
| Xiang Tu | PWR | - | Zhejiang | FHPRI | SE | B |
| Xiao Bai Tao | PWR | Local Cultivar | Henan | NPGR(Zhengzhou) | N | L |
| Xiao Hong Hua | PWR | Local Cultivar | Jiangsu | NPGR(Jiangsu) | SE | L |
| Xiao Jin Dan | PYR | - | Yunnan | NPGR(Zhengzhou) | NW | L |
| Xin Bai Hua | PWR | Seedling Selection of Bai Hua Shui Mi | Jiangsu | NPGR(Jiangsu) | SE | B |
| Xin Duan Yang | PWR | - | unknown | NPGR(Zhengzhou) |  | L |
| Xin Hong | PWR | - | Chengdu | FHPRI | SW | L |
| Xin Jiang Huang Rou Tao | PYR | Local Cultivar | Xingjiang | NPGR(Zhengzhou) | NW | L |
| Xing Yu | PWR | - | Zhejiang | FHPRI | SE | B |
| Xu Mi | PWR | Seedling Selection of Bai Hua | Jiangsu | FHPRI | SE | B |
| Xue Xiang Lu_FH | PWR | Bai Hua Shui Mi×Hatsukami | Beijing | NPGR(Jiangsu) | N | B |
| Xue Xiang Lu_NJ | PWR | Bai Hua Shui Mi×Hatsukami | Beijing | NPGR(Jiangsu) | N | B |
| Yahata Hakuto | PWF | - | Japan | FHPRI | J | L |
| Yamanashi Hakuho | PWR | Hakuho Mutation | Japan | FHPRI | J | B |
| Yamato Hakuto | PWR | Hakuto × Carmen | Japan | SAAS | J | B |
| Yan Feng | PWR | - | Zhejiang | FHPRI | SE | B |
| Yan Guang | NWR | Rui Guang3×Armking | Chongqing | SWU | SW | B |
| Yan Hong | PWR | Seedling Selection of Lv Hua9 | Beijing | NPGR(Jiangsu) | N | B |
| Yan Hong11 | NWR | - | Beijing | SWU | N | L |
| Yan Wo Hong | PWR | - | Hebei | NPGR(Jiangsu) | N | L |
| Yan Xia | NWR | Okubo×Okitsu | Shanxi | SAAS | N | B |
| Yang Qu Bai Tao | PWR | Local Cultivar | Shanxi | SAAS | N | L |
| Yang Shan2 | PWR | - | Jiangsu | NPGR(Jiangsu) | SE | L |
| Yangzhou3 | PWR | - | Jiangsu | NPGR(Zhengzhou) | SE | L |
| Ye Mao Tao | PWR | - | Shanxi | SAAS | N | WL |
| Ye Sheng Tao | PWR | - | Zhejiang | NPGR(Zhengzhou) | SE | L |
| Yi Pin Hong | PWR | - | chongqing | SWU | SW | L |
| Yi Xian Hong | PWR | Local Cultivar | Beijing | NPGR(Zhengzhou) | N | L |
| Ying Guang You Tao | NWR | - | Zhejiang | NPGR(Zhengzhou) | SE | L |
| Ying Qing | PWR | - | Jiangsu | NPGR(Jiangsu) | SE | B |
| Ying Qiu | PWR | - | Chongqing | SWU | SW | L |
| Ying Zui Tao | PWR | Local Cultivar | Anhui | NPGR(Zhengzhou) | SE | L |
| You Ming | PWR | You Ming Bai Tao Mutation | Korea | SWU | K | B |
| You Ming Bai Tao | PWR | Okubo×Numome | Korea | FHPRI | K | B |
| You Pan | NWF | - | Gansu | SAAS | NW | L |
| Yu Ci Bai Tao | PWR | - | Shanxi | Shanxi | N | L |
| Yu Hua Lu | PWR | Bai Hua Shui Mi ×Shang Hai Shui Mi | Zhejiang | NPGR(Jiangsu) | SE | B |
| Yu Lu | PWR | Lao Yu Lu Mutation | Zhejiang | FHPRI | SE | L |
| Yu Lu Pan Tao | PWF | - | Zhejiang | FHPRI | SE | L |
| Yu You1 | PWR | - | Chongqing | SWU | SW | L |
| Yu You2 | NWR | - | Chongqing | NPGR(Zhengzhou) | SW | L |
| Yuan Chun Bai | PWR | Local Cultivar | Hebei | NPGR(Jiangsu) | N | L |
| Yuan Dong Bai Tao_JS | PWR | Seedling Selection from Lian Huang | Zhejiang | NPGR(Jiangsu) | SE | B |
| Yuan Dong Bai Tao_SX | PWR | Seedling Selection from Lian Huang | Zhejiang | NPGR(Jiangsu) | SE | B |
| Yue165 | PWR | - | Guangdong | NPGR(Jiangsu) | S | L |
| Yue192 | PWR | - | Guangdong | NPGR(Jiangsu) | S | L |
| Yuzora | PWR | Hakuto×Akatsuki | Japan | FHPRI | J | B |
| Zao Feng | PWR | Bai wa×Tachibana Wase | Dalian | NPGR(Zhengzhou) | NE | B |
| Zao Feng Huang | PWR | - | unknown | NPGR(Zhengzhou) |  | L |
| Zao Feng Wang | PWR | Bud Mutant of Zao Feng | Hebei | NPGR(Jiangsu) | N | B |
| Zao Hong Bao Shi | NWR | - | Henan | FHPRI | N | L |
| Zao Hong Xia | NWR | Armking× (Jing Yu×NJN76) | Beijing | NPGR(Zhengzhou) | N | B |
| Zao hong yan | NYR | Mutant from Yu Hua Lu | Jiangsu | NPGR(Zhengzhou) | SE | B |
| Zao hong zhu | NWR | Jing Yu× A369 | Beijing | NPGR(Zhengzhou) | N | B |
| Zao Hua Lu | PWR | Seedling Selection of Yu Hua Lu | Jiangsu | NPGR(Jiangsu) | SE | B |
| Zao Huang Guan | PYR | Seedling Selection of Zao Sheng Huang Jin | Shan'xi | NPGR(Zhengzhou) | NW | B |
| Zao Huang Jin | PYR | Local Cultivar | Beijing | NPGR(Zhengzhou) | N | L |
| Zao Huang Pan Tao | PYF | 8-21 Pan Tao×Flat peach | Zhengzhou | NPGR(Zhengzhou) | N | B |
| Zao Huang Tao | PYR | - | unknown | NPGR(Zhengzhou) |  | L |
| Zao jiu bao | PWR | Local Cultivar | unknown | NPGR(Zhengzhou) | N | L |
| Zao Kui | PWR | - | unknown | NPGR(Zhengzhou) |  | L |
| Zao Lu Pan Tao_ZZ | NWF | Sa Hua Hong Pan Tao×Zao Xiang Yu | Beijing | NPGR(Zhengzhou) | N | B |
| Zao Mei | PWR | Qing Feng × Zhao Xia | Beijing | NPGR(Jiangsu) | N | B |
| Zao Pan42 | NWF | Bud mutatant from Zao Lu Pan Tao | Shanxi | SAAS | N | B |
| Zao Shang Hai Shui Mi | PWR | Unknown | Shanghai | NPGR(Jiangsu) | SE | L |
| Zao Shu Da Jiu Bao | PWR | Okubo Mutation | Shanxi | SAAS | N | B |
| Zao Shu Li He Yu Lu | PWR | - | Zhejiang | NPGR(Jiangsu) | SET | L |
| Zao Shu You Ming | PWR | Unknown | unknown | SWU | - | B |
| Zao Tai Mi | PWR | - | unknown | FHPRI | - | L |
| Zao Xia Lu | PWR | Sunagowase × Yu Hua Lu | Zhengzhou | NPGR(Jiangsu) | SE | B |
| Zao Xiang Yu | PWR | Okubo × Hatsukami | Beijing | NPGR(Jiangsu) | N | B |
| Zao Zhen Bao | PWR | - | Zhejiang | FHPRI | SE | B |
| Zao Zhong You4 | NYR | Mutant of Zhong You 4 | Zhengzhou | NPGR(Zhengzhou) | N | B |
| Zhangbai 2 | PWR | Local Cultivar | Gansu | NPGR(Zhengzhou) | NW | L |
| Zhangbai5 | PWR | - | Gansu | NPGR(Zhengzhou) | NW | L |
| Zhao Hui | PWR | Bai Hua Shui Mi × Tachibanawase | Jiangsu | NPGR(Jiangsu) | SE | B |
| Zhao Xia | PWR | Bai Hua×Hatsukami | Jiangsu | NPGR(Jiangsu) | SE | B |
| Zhen Zhou7_CQ | PWR | Hakuho×Bi Tao | Zhengzhou | SWU | N | B |
| Zheng Huang 2 | PYR | Guan Tao5×Feng Huang | Zhengzhou | NPGR(Zhengzhou) | N | B |
| Zheng Huang 3 | PYR | Zao Shu Huang Gan×Feng Huang | Zhengzhou | NPGR(Zhengzhou) | N | B |
| Zheng huang 4 | PYR | Zao Shu Huang Gan×Feng Huang | Zhengzhou | NPGR(Zhengzhou) | N | B |
| Zheng1-39 | PWR | - | Zhengzhou | SWU | N | B |
| Zheng1-45 | PWR | - | Zhengzhou | SWU | N | B |
| Zheng15-1 | PYR | - | Zhengzhou | NPGR(Zhengzhou) | N | B |
| ZhengZhou 11 | PWR | Hakuho×Bi Tao | Zhengzhou | NPGR(Zhengzhou) | N | B |
| Zhengzhou 7_ZZ | PWR | Hakuho×Bi Tao | Zhengzhou | NPGR(Zhengzhou) | N | B |
| ZhengZhou 8 | PWR | Hakuho×Bi Tao | Zhengzhou | NPGR(Zhengzhou) | N | B |
| Zhong Hua Shou Tao | PWR | Dongtao Bud Mutation | Shandong | SAAS | N | B |
| Zhong Qiu Mi | PWR | - | Heilongjiang | FHPRI | NE | L |
| Zhong Shan Zao Lu | PWR | Bai Hua Shui Mi × Hatsukami | Jiangsu | SAAS | SE | B |
| Zhong You 4 | NYR | 25-17×May Fire | Zhengzhou | NPGR(Zhengzhou) | N | B |
| Zhong You 5_FH | NWR | 25-10×May Fire | Zhengzhou | NPGR(Zhengzhou) | N | B |
| Zhong You 5_ZZ | NWR | 25-11×May Fire | Zhengzhou | NPGR(Zhengzhou) | N | B |
| Zhong You 8 | NYR | Hong Shan Hu xQing Lang | Zhengzhou | NPGR(Zhengzhou) | N | B |
| Zhong You Pan 2 | NWF | - | Zhengzhou | NPGR(Zhengzhou) | N | B |
| Zhou Ye Huang Lu | PYR | Local Cultivar | Zhejiang | NPGR(Jiangsu) | SE | L |

Note:^a^: NPGR means National Peach Germplasm Repositories; SAAS means Shanxi Academy of Agriculture Sciences; FHPRI means Fenghua Peach Research Institute; SWU means Southwest University

^b^: the first alphabet indicate fruit shape: peach (P), nectarine (N), the second alphabet indicate flesh color: white (W), yellow(Y), red(R) , the third alphabet indicate shape: flat peach (F) and round peach(R)

^c^: indicate the different zone located in China, N: North; NW: Northwest; NE: Northeast; S: South; SE: Southeast; SW: Southwest

^d^: Breeding/landrace: B means breeding, L means Landrace, W means wild relative species
